# Transcriptional Responses in Root and Leaf of *Prunus persica* under Drought Stress Using RNA Sequencing

**DOI:** 10.3389/fpls.2016.01715

**Published:** 2016-11-23

**Authors:** Najla Ksouri, Sergio Jiménez, Christina E. Wells, Bruno Contreras-Moreira, Yolanda Gogorcena

**Affiliations:** ^1^Department of Pomology, Estación Experimental de Aula Dei-Consejo Superior de Investigaciones CientíficasZaragoza, Spain; ^2^Department of Biological Sciences, Clemson University, ClemsonSC, USA; ^3^Laboratory of Computational and Structural Biology, Department of Genetics and Plant Production, Estación Experimental de Aula Dei – Consejo Superior de Investigaciones CientíficasZaragoza, Spain; ^4^Fundación ARAIDZaragoza, Spain

**Keywords:** GF677, Catherina, RNA sequencing, leaf, root, rootstock, drought, peach

## Abstract

*Prunus persica* L. Batsch, or peach, is one of the most important crops and it is widely established in irrigated arid and semi-arid regions. However, due to variations in the climate and the increased aridity, drought has become a major constraint, causing crop losses worldwide. The use of drought-tolerant rootstocks in modern fruit production appears to be a useful method of alleviating water deficit problems. However, the transcriptomic variation and the major molecular mechanisms that underlie the adaptation of drought-tolerant rootstocks to water shortage remain unclear. Hence, in this study, high-throughput sequencing (RNA-seq) was performed to assess the transcriptomic changes and the key genes involved in the response to drought in root tissues (GF677 rootstock) and leaf tissues (graft, var. Catherina) subjected to 16 days of drought stress. In total, 12 RNA libraries were constructed and sequenced. This generated a total of 315 M raw reads from both tissues, which allowed the assembly of 22,079 and 17,854 genes associated with the root and leaf tissues, respectively. Subsets of 500 differentially expressed genes (DEGs) in roots and 236 in leaves were identified and functionally annotated with 56 gene ontology (GO) terms and 99 metabolic pathways, which were mostly associated with aminobenzoate degradation and phenylpropanoid biosynthesis. The GO analysis highlighted the biological functions that were exclusive to the root tissue, such as “locomotion,” “hormone metabolic process,” and “detection of stimulus,” indicating the stress-buffering role of the GF677 rootstock. Furthermore, the complex regulatory network involved in the drought response was revealed, involving proteins that are associated with signaling transduction, transcription and hormone regulation, redox homeostasis, and frontline barriers. We identified two poorly characterized genes in *P. persica*: growth-regulating factor 5 (GRF5), which may be involved in cellular expansion, and AtHB12, which may be involved in root elongation. The reliability of the RNA-seq experiment was validated by analyzing the expression patterns of 34 DEGs potentially involved in drought tolerance using quantitative reverse transcription polymerase chain reaction. The transcriptomic resources generated in this study provide a broad characterization of the acclimation of *P. persica* to drought, shedding light on the major molecular responses to the most important environmental stressor.

## Introduction

*Prunus persica* L. Batsch, or peach, a deciduous species from the family *Rosaceae*, is among the most prevalent commercially grown perennial fruit trees worldwide, ranking third after apple and pear trees (FAOSTAT, 2016)^1^. Originating from China, the peach tree is also a model species among woody plants due to its self-compatibility, short juvenile phase (2–3 years), and relative genetic simplicity (2n = 2x = 16), with approximately 230 Mbp (Arús et al., 2012). The majority of stone fruit trees, and especially the peach, are usually grafted on to rootstocks that belong to either the same species or other *Prunus* species (Rodríguez-Celma et al., 2013). In fact, rootstocks are an essential component in modern fruit production as they can adapt to diverse environmental conditions and cultural practices (Jiménez et al., 2013).

Among the environmental factors that present threats to agricultural production, drought has the largest impact, as it decreases crop productivity more than any other environmental factor (Walter et al., 2011). In order to withstand low levels of water availability, plants have evolved various strategies: (i) escape (by completing their life cycle before the onset of acute drought), (ii) avoidance (by relying on morphological changes to retain sufficient water), and (iii) drought tolerance (by producing osmo-protectants, which relieve the negative impact of dehydrative stress) (Jarzyniak and Jasiński, 2014). Most climate projections based on the continuation of current global situation forecast droughts of increased severity (Walter et al., 2011). Therefore, increasing drought tolerance is currently a major goal of *Prunus* rootstock breeding programs as water deficits influence a wide range of plant processes, at both the molecular level and the morphological level (Arismendi et al., 2015). A deeper understanding of the molecular basis of drought tolerance in *Prunus* rootstocks and the identification of genes involved in the response to this stress is a key step toward improving the drought tolerance of stone fruit trees using advanced marker-assisted selection (MAS).

Over the last decade, studies conducted using cDNA amplified fragment length polymorphism (cDNA-AFLP), expressed sequence tag (EST) sequencing, and microarrays have highlighted that plants in general, and *Prunus* spp. in particular, have developed a range of physiological and molecular responses to cope with soil water scarcity (Shinozaki and Yamaguchi-Shinozaki, 2006; Martínez-Gómez et al., 2011). Despite their value, these approaches have several important limitations. In fact, EST technology has been hampered by its low coverage, high error rate and high cost while the microarray-based analysis faces the constraints of specific probe design and RNA-variants detection (Valdés et al., 2013). As an alternative, current RNA sequencing (RNA-seq) technologies offer increased specificity and sensitivity for the enhanced detection of genes, transcripts, and differential expression profiling, which has permitted gene function to be monitored at the level of the entire genome (Wang et al., 2013). Combined with bioinformatics, high-throughput RNA-seq offers more opportunities to identify novel genes related to specific traits and it has enabled us to better understand transcriptomic changes both during environmental perturbation (Martin et al., 2013) and during biotic attack (Rubio et al., 2014). However, the analysis of RNA-seq must be done with great care, as it is not straightforward (Conesa et al., 2016).

The aim of this study is to shed light on the complex molecular mechanisms that underlie the responses of *Prunus* spp. to water deficits. In order to achieve this goal, Illumina’s HiSeq 2000 Sequencing System was used to sequence the RNA in both the roots of GF677 rootstock and the leaves of a graft of the Catherina cultivar. This allowed the transcriptomic variations among drought-stressed plants and a control group to be explored, and the differentially expressed genes (DEGs) that may be associated with drought tolerance in *P. persica* to be identified. In addition to the transcriptome analysis, gene expression profiling was carried out and functional annotations were added to provide an overview of the drought acclimation process. Together with the use of quantitative reverse transcription polymerase chain reaction (RT-qPCR) to validate the results from the RNA-seq analysis, the results provide an overview of the changes in the *Prunus* spp. transcriptome that are triggered by drought stress.

In contrast to other studies, this work represents the first characterization of drought-related genes in *P. persica* that involves assessing both roots and leaves at the same time. These tissues were chosen as the roots are the first plant tissue to perceive drought stress, while leaves are central to the control of water loss (Garg et al., 2016). Our data contribute to the understanding of drought responses in plants and serve as a publicly available resource for future gene expression, genomic, and functional studies of *Prunus* spp.

## Materials and Methods

### Plant Material and Drought Stress Experiment

Clonally propagated plants from the GF677 rootstock (*Prunus dulcis* Miller ×*P. persica*), which was selected for its high level of drought tolerance, were acquired from a commercial nursery (Agromillora Iberia, S.L., Barcelona, Spain). The rootstocks were grown for 2 weeks in 300 cm^3^ pots containing a peat substrate, and then they were micrografted with *P. persica* var. Catherina.

Subsequently, 30 representative plants were transplanted into 15 L containers with TKS-1, a 1:1 ratio of sand to peat substrate (Floragard, Oldenburg, Germany) and 2 g kg^-1^ Osmocote 14-13-13 (The Scotts Company LLC, Maryville, OH, USA). The plants were grown in an experimental greenhouse in Zaragoza, Spain (41°43′ N, 0°48′ W) under controlled environmental conditions (23°C day/18°C night, 14 h light/10 h dark photoperiod) for 21 days before the start of the experiment. During this period (April 2011 to May 2011) the plants were watered daily until runoff was visible.

The drought stress experiment started on May 14 and continued for 16 days. The 30 plants were randomly separated into two groups: well-watered plants (the control plants) and water-deprived plants (the drought-stressed plants). The control plants were watered daily to field capacity while the stressed plants were watered with 80% of the quantity of water that had evapotranspired the previous day (Marcelis et al., 2007). The soil water content was measured using time domain reflectometry (TDR), with 20 cm-long probes inserted vertically into the containers, as described in Moret-Fernández et al. (2012). The soil water content and physiological parameters, namely, stem water potential (Ψs), stomatal conductance (*g_s_*), photosynthetic rate (*A_N_*), and intercellular CO_2_ concentration (*Ci*), were recorded for both groups. These measurements were taken on days 0, 7, 12, and 16 after the start of the experiment, on clear days between 10:00 and 12:00, as reported in Jiménez et al. (2013). On day 16, leaf and root samples of three randomly selected biological replicates were collected from both the control and drought-stressed plants (12 samples in total). The plant tissues were then frozen in liquid nitrogen and stored at -80°C.

### RNA Extraction

The total RNA from the three biological replicates was extracted from the root and leaf samples of the control and water-deprived plants according to the method described by Meisel et al. (2005), which was adapted to mini-preparations (Jiménez et al., 2013). Subsequently, samples were treated with DNase I (Thermo Fisher Scientific, Waltham, MA, USA) to remove the contaminating genomic DNA. RNA integrity and purity were assessed using the NanoDrop 2000 Spectrophotometer (Thermo Fisher Scientific) and the Agilent 2100 Bioanalyzer (Agilent Technologies, Santa Clara, CA, USA). Only RNA samples with A260/A280 ratios from 1.9 to 2.1, A260/A230 ratios ≥ 2, and RNA integrity values > 8 were used in the subsequent experiments.

### RNA Next Generation Sequencing

**FIGURE 1:**
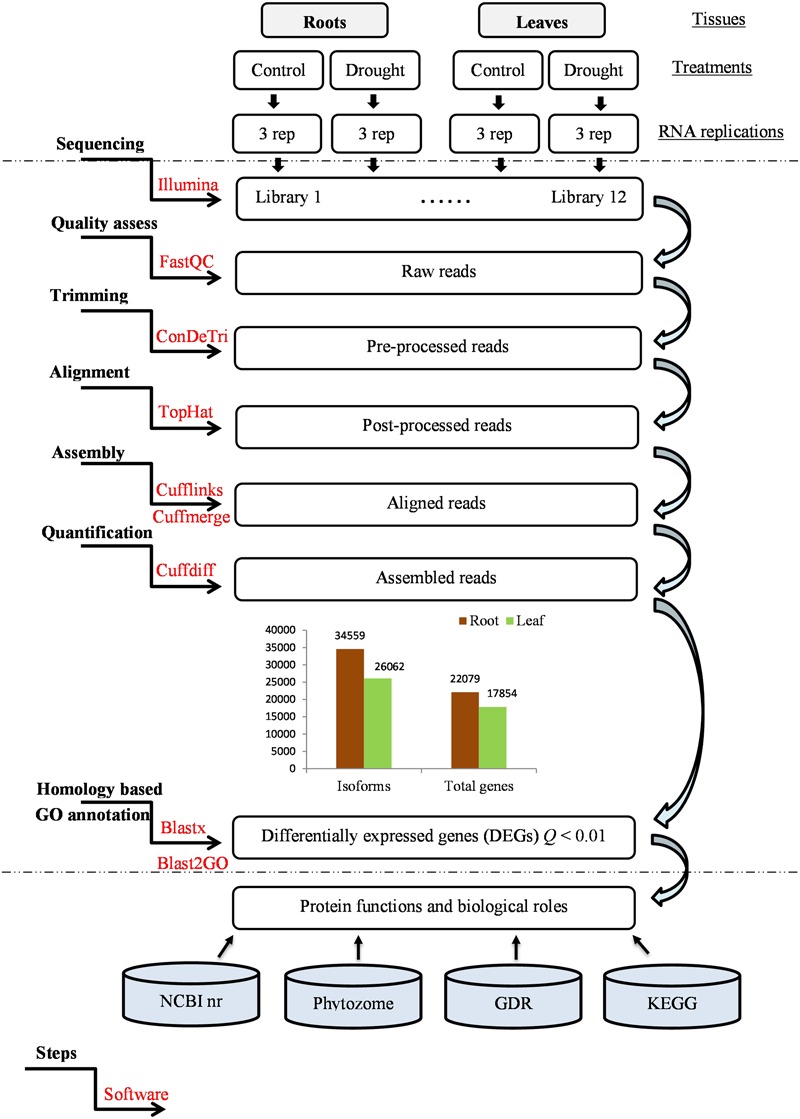
**RNA-seq pipeline**.

Equal amounts of total RNA of each tissue from each experimental group (see **Figure 1**) were used to construct 12 RNA libraries. Total RNA was submitted to Otogenetics Corporation (Atlanta, GA USA) for RNA-Seq assays. Briefly, 1–2 μg of cDNA was generated using Clontech Smart cDNA kit (Clontech Laboratories, Inc., Mountain View, CA, USA, catalog# 634925) from 100 ng of total RNA. cDNA was fragmented using Covaris (Covaris, Inc., Woburn, MA, USA), profiled using Agilent Bioanalyzer, and subjected to Illumina library preparation using NEBNext reagents (New England Biolabs, Ipswich, MA, USA, catalog# E6040). The quality and quantity and the size distribution of the Illumina libraries were determined using an Agilent Bioanalyzer 2100 (Agilent Technologies). The libraries were then submitted for Illumina HiSeq2000 sequencing according to the standard operation. Paired-end 90–100 nucleotide (nt) reads were generated and checked for data quality using FASTQC (Babraham Institute, Cambridge, UK). FASTQ file was sent to customer for downstream analysis. All the raw reads data were deposited with the European Nucleotide Archive (ENA) as part of project PRJEB12334.

### Transcript Assembly

The quality of the raw paired-end reads was monitored using FastQC v0.10.0. Low quality segments, erroneous base calls, or adapter fragments were trimmed using ConDeTri Perl script v5.8.9, which discarded data with poor quality scores (*Q* < 25) or read lengths < 35 bp (Smeds and Kunstner, 2011). Post-processed reads were mapped to the *P. persica* Whole Genome Assembly v1.0 using TopHat software v2.0.3. This software was selected as a mapping tool because it can generate a database of splice junctions based on the gene model annotation (Trapnell et al., 2012). The TopHat parameters were set at < 3 mismatches when mapping reads and a maximum of 20 multiple hits for each library. The resulting aligned sequences were used with the Cuﬄinks suite v1.3.0. In order to generate assembled transcripts for each tissue type and each experimental group. These assemblies were then merged together using the Cuffmerge tool to provide a uniform basis for calculating transcript expression. All the assembled transcripts were deposited with the ENA under accession numbers HADJ01000001–HADJ01060423.

### Quantification of the Levels of Gene Expression and Differential Expression Analysis

The changes in the relative abundance of transcripts in drought versus control conditions were quantified and normalized to the number of reads per kilobase of transcripts per million mapped reads (RPKM). The gene expression levels were then estimated using the Cuffdiff program from the Cuﬄinks suite. The statistical significance of the level of differential expression of each gene in the roots and leaves was determined by initially setting the false discovery rate (FDR)-adjusted *P*-values at 0.05 (which are known as *Q*-values), using the Benjamini and Hochberg method (Benjamini and Hochberg, 1995). However, in order to reduce the likelihood of false positives, only DEGs with a *Q*-value < 0.01 were considered for further analysis. The complete workflow of the RNA-seq analysis is provided in **Figure 1**.

### Homology Search and Functional Annotation

Significant DEGs were annotated using BLASTX by scanning four standard resources: the NCBI nr database^2^, Phytozome11^3^, the Genome Database of Rosaceae (GDR)^4^, and the Kyoto Encyclopedia of Genes and Genomes (KEGG). The E-value cut-off was set to 1E-5 and a 70% query coverage threshold was used to discard partial/single-domain protein matches. A gene ontology (GO) analysis was performed using standalone Blast2GO v3.2 with the same E-value cut-off. This software assigned GO terms to each DEG to allow their putative functions to be predicted in terms of molecular functions (MFs), biological processes (BPs), and cellular components (CCs) (Gotz et al., 2008). These annotations were enhanced by merging them with InterProScan-assigned GO terms, and then running the annotation augmentation module (Annex). The resulting GO terms were plotted and visualized with the Web Gene Ontology Annotation Plot (WEGO) tool^5^ (Ye et al., 2006).

### Gene Ontology (GO) Enrichment

Gene ontology enrichment was carried out for the DEGs associated with both tissues using the singular enrichment analysis (SEA) function of the web-based tool AgriGO^6^. The input list consisted of the whole set of DEGs, while the annotation of Peach Genome (v1.0 Joint Genome Institute) was used as a pre-computed background. Overrepresented terms in the three main categories (BP, MC, and CC) were filtered using Fisher’s exact test and the Benjamini-Hochberg multiple testing correction (*Q-*value < 0.05).

### RT-qPCR Validation

In order to confirm the reliability and accuracy of the RNA-seq analysis, RT-qPCR was performed on a set of 34 root and leaf genes selected for their putative drought-related functions, including 16 upregulated genes (Log_2_FC > 2), seven unchanged genes (|Log_2_FC| < 2), and 10 downregulated genes (Log_2_FC < -2). In these expressions, FC is the fold change ratio between the drought-stressed and control group RPKM expression (Supplementary Table S1).

The total RNA samples (1.5 μg) were treated with DNase I to remove the contaminating genomic DNA. Subsequently, the samples were reverse transcribed using oligo (dT)_18_ as a primer with RevertAid H Minus First Strand cDNA Synthesis Kit (Thermo Scientific). The RT-qPCR reactions were performed with the 7500 Fast Real-Time PCR System v2.0.1 (Applied Biosystem by Life Technologies, Grand Island, NY, USA) using bi-technical replicates and tetra-biological replicates for each tissue-experimental group (two of the biological replicates were from the same plants used in the RNA-seq analysis). The reactions were performed using 10 μl of SYBR Green Master Mix (Kapa Biosystems, Boston, MA, USA), 1 μl of each primer (making a total of 4 μM), and 5 μl of diluted cDNA in a final volume of 20 μl. Control cDNA and control primer were included for each run. The primers were designed using NCBI primer-BLAST software^7^ according to the following criteria: primer size of 18–22 bp, GC content between 40 and 60%, amplicon size of 90–160 bp, and annealing temperatures from 57 to 62°C. Moreover, the primers were aligned to the target gene sequence using BioEdit software v7.2 to ensure specific annealing. The likely secondary structures were also assessed to avoid hairpins and primer dimers. A BLASTN scan of the theoretical amplicon was carried out to test the homology to the target genes. Finally, each of the products underwent gel electrophoresis to confirm the presence of a single amplicon of the expected length. The primer sequences and features are listed in Supplementary Table S1. The efficiencies and quantification cycle (Cq) for each gene were calculated using the LinRegPCR program (Ruijter et al., 2009). Gene expression measurements were determined using the gene expression difference (GED) formula (Schefe et al., 2006). Actin 2 and AGL-26 were used as reference genes for data normalization. The relative expression was calculated with respect to the GF677 rootstock control group. A correlation analysis between the levels of gene expression according to the RNA-seq and RT-qPCR analyses was performed using SPSS v23.0.

## Results

### Phenotypic and Physiological Response to Drought Stress

After a period of drought stress of 16 days, the main visible effects on the plants were wilting and slight defoliation due to the decreased turgor pressure and the shrinkage of the leaf cells. In order to verify that the visual symptoms were indicative of exposure to water deficit conditions, the soil water content and standard physiological parameters of drought-induced effects on leaves were measured (**Table 1**). The soil water content dropped remarkably from 26.63% in the control plants to 10.69% in the drought-stressed plants, which indicated the presence of decreased turgor pressure and therefore explained the wilting. A decrease was also observed for the stem water potential (Ψs) in response to the reduction in soil water content, further confirming that the plants experienced drought stress. In addition, as leaf water status is considered to be a reliable indicator of plant water balance, stomatal conductance (*g_s_*), intercellular CO_2_ concentration (*Ci*), and the net photosynthetic rate (*A_N_*) were measured. The results revealed that the drought-stressed plants exhibited a lower stomatal conductance compared to the control plants. This reduction led to a significant decline in intracellular CO_2_ concentrations, which decreased from 277.30 μmol CO_2_ mol^-1^ to 261.27 μmol CO_2_ mol^-1^. Taken together, these results explain the observed slowdown of the photosynthetic machinery (*A_N_*) as a result of the drought conditions.

**Table 1:** Soil water content (SWC), stem water potential (Ψs), stomatal conductance (*g_s_*), CO_2_ concentration (*Ci*), and photosynthetic rate (*A_N_*) in leaves (graft, var. Catherina) in control and drought-stressed plants after 16 days.

Treatments	SWC %	Ψs MPa	*g_s_* mol H_2_O m^-2^ s^-1^	*Ci*μmol CO_2_ mol^-1^	*A_N_*μmol CO_2_ m^-2^ s^-1^
Well-watered	26.63 ± 0.18	-0.73 ± 0.05	0.56 ± 0.03	277.30 ± 2.17	20.82 ± 0.54
Water-deprived	10.69 ± 0.28	-1.08 ± 0.02	0.37 ± 0.03	261.27 ± 4.27	18.35 ± 0.42

*Data are mean ± SE of *n* = 6 replicates. MPa, megapascal. The means were compared using t-Student tests and were found to be significant in all cases (*P <* 0.05).*

### RNA-Sequencing and Transcriptomic Profiles

As roots are the first organs to be exposed to drought, and leaves are the first to sense water loss, both tissues were sampled from the control and the drought-stressed plants and used for transcriptome analysis to obtain an overview of the responses of *P. persica* during water deprivation. Three biological replicates were processed in order to construct 12 RNA libraries (**Figure 1**). A total of 315 M paired 100 bp reads were generated, ranging from 20.81 to 61.30 M raw reads per library. Among those, more than 188 M (59.85%) high-quality sequences were retained after pre-processing and filtering out reads containing adaptors, short reads (<35 bp), and reads with low quality scores (*Q* < 25). The remaining 136 M single and paired reads (72.34% of all the high-quality sequences) were mapped to the *Prunus* reference genome (from 10.18 to 14.78 M reads per library), which showed that the quality of these mapped genes was sufficient to conduct the subsequent analysis. A summary of the raw data generated, and the trimmed and mapped reads, is summarized in **Table 2**.

**Table 2:** Summary of RNA sequencing data in million (M) reads from 12 RNA libraries of control and drought-stressed roots (GF677 rootstock) and leaves (graft, var. Catherina) after 16 days of drought.

Libraries	Raw reads (M)	Clean reads (M)			Mapped reads (M)		
		Paired	Unpaired	Total	Paired	Unpaired	Total
RC 1	61.30	28.54	12.31	40.85	10.71	4.16	14.78
RC 2	25.52	10.10	5.15	15.25	7.83	4.07	11.90
RC 3	22.69	8.48	4.75	13.23	7.03	3.95	10.98
RD 1	24.97	9.37	5.04	14.41	7.49	4.05	11.54
RD 2	24.49	7.76	5.47	13.23	5.89	4.29	10.18
RD 3	20.81	8.14	4.79	12.93	6.51	3.81	10.32
LC 1	23.12	8.72	5.44	14.16	7.14	4.57	11.71
LC 2	23.02	9.05	5.30	14.35	7.48	4.51	11.99
LC 3	22.30	8.01	4.76	12.77	6.63	4.07	10.70
LD 1	22.00	8.33	4.42	12.75	6.77	3.75	10.84
LD 2	22.86	6.20	5.51	11.71	5.37	4.93	10.30
LD 3	22.00	8.63	4.33	12.96	7.20	3.78	10.38
Total	315.08	121.33	67.27	188.60	86.05	49.94	135.99

*RC, root control; RD, root drought-stressed; LC, leaf control; LD leaf drought-stressed.*

Consequently, the aligned sequence reads were used for reference-guided assembly and thereafter merged using the Cuﬄinks–Cuffmerge workflow. Overall, 34,559 and 26,062 transcript isoforms were obtained from roots and leaves, respectively, which correspond to 22,079 and 17,854 genes, respectively (**Figure 1**).

### Analysis of DEGs

**FIGURE 2:**
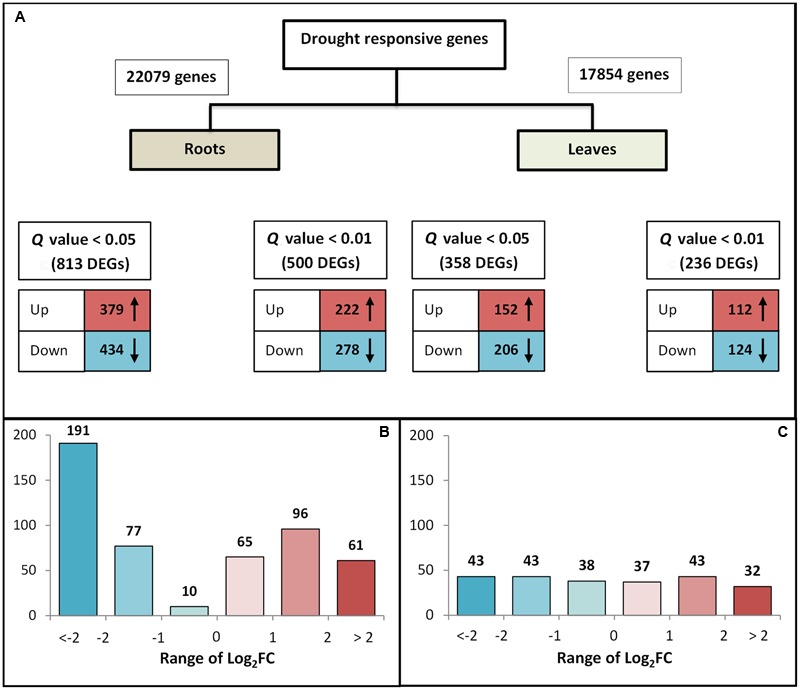
**(A)** Number of total and differentially expressed genes (DEGs) showing either upregulation or downregulation at different *Q*-values in roots (GF677 rootstock) and leaves (graft, var. Catherina). Up: upregulated genes (red); down: downregulated genes (blue). **(B,C)** Relative expression of DEGs selected at *Q*-value < 0.01 in roots and leaves. The color intensity indicates the level of the change in expression: a darker color represents a larger change in expression. The *x*-axis indicates the range of Log_2_FC. The fold change (FC) was calculated as the ratio between the drought-stressed and control plants, while the *y*-axis indicates the number of detected DEGs.

In order to explore the transcriptional response to drought stress, genes in both roots and leaves were tested for differential expression between the control and drought conditions. Expression levels for each gene were calculated and normalized to RPKM values. Initially, the multiple testing correction involved a *Q*-value < 0.05 and a total of 1,171 genes were found to exhibit differential expression (813 in the roots and 358 in the leaves). Subsequently, a more stringent *Q-*value was applied (< 0.01) in order to identify the most reliable DEGs. In this analysis, 500 DEGs were identified in the roots and 236 in the leaves. The distribution of these genes is provided in **Figure 2**. As illustrated, in drought stress conditions, the number of downregulated genes was slightly higher than the number of upregulated genes. Furthermore, the analysis revealed that there were approximately twice the number of DEGs in the roots than in the leaves, confirming the expectations that (i) the root is the first organ that senses and is affected by drought stress and (ii) roots respond faster to stress than leaves, undergoing more complex gene regulation during water deprivation. These results further highlight the key role of rootstocks as stress buffers.

### Annotation of DEGs

In order to assign putative functions to the DEGs, all identified transcripts with a *Q*-value < 0.01 were subjected to Blast2GO annotation. After enhancing the annotation by running InterProScan and Annex, 1,904 root annotations and 815 leaf annotations were obtained. Out of the 500 DEGs in the roots, 361 (72.2%) were successfully annotated; however, 52 (10.4%) DEGs did not match any of the sequences characterized in the databases, which may indicate the presence of novel genes. Among the root sequences, 53 (14.7%) were either hypothetical or uncharacterized proteins. Regarding the leaves, 169 out of 236 sequences (71.6%) were annotated, including 19 (11.2%) with hypothetical or uncharacterized functions, while 34 (14.4%) sequences had no significant hits. A summary is presented in Supplementary Figure S1. Regarding the matched species, the majority of the highest-scoring hits were from *P. persica* (70.49% in the root and 79.69% in the leaves) and *P. mume* (24.63% in the roots and 16.75% in the leaves), which belongs to the family Rosaceae. These results confirm the quality of our data and the assembly process. A graph displaying the species distribution and the top BLASTX hits is provided in Supplementary Figure S2.

**FIGURE 3:**
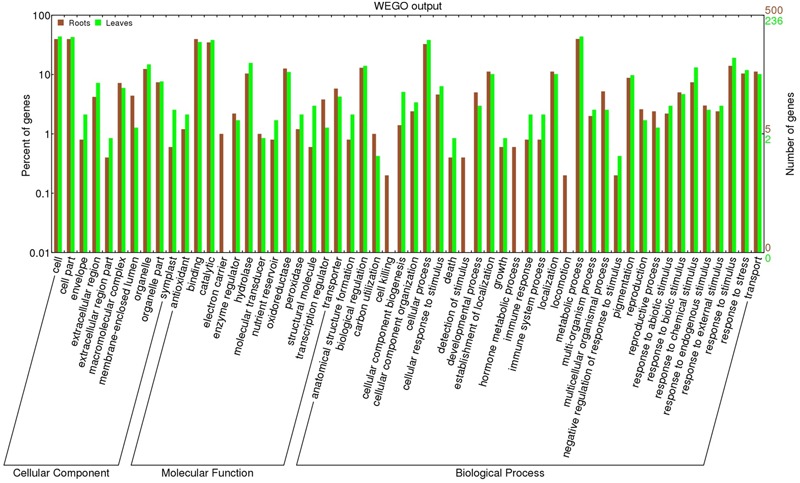
**Histogram of GO terms assigned to DEGs in roots (GF677 rootstock, *n* = 500) and leaves (graft, var. Catherina, *n* = 236).** The DEGs are categorized into three main groups: cellular components (CCs), molecular functions (MFs), and biological processes (BPs). Note that the vertical axes use a logarithmic scale.

The entire set of DEGs was subjected to GO analysis in order to achieve a broader functional characterization. As a result, 500 DEGs in the roots and 236 in the leaves were classified into 56 subcategories within three main categories (BP, MF, and CC). In total, 283 DEGs in the roots and 137 in the leaves were associated with BP terms, 298 root DEGs and 139 leaf DEGs were associated with MF terms, and 211 root and 108 leaf DEGs were annotated with CC terms (**Figure 3**). Note that in many cases the same sequence can be assigned to more than one category. In both tissues, the most represented BP subcategories were “metabolic process,” followed by “cellular process” and “response to stimulus” (Supplementary Figure S3). As for MF, the major subcategories were “binding” and “catalytic activity”. Finally, among the CC terms, “membrane” was the most dominant subcategory for the roots, followed by “cell” and “cell part”, which were associated with the leaves. These results, with a comprehensive list of GO subcategories, are plotted in **Figure 3**.

The annotations within the BP category were the most informative, as they are easier to interpret in the context of drought responses. The list of DEGs annotated with “metabolic process” is an important resource for the identification of novel genes involved in drought acclimation. In addition, **Figure 3** shows that “locomotion” (GO: 0040011), “hormone metabolic process” (GO: 0042445), “detection of stimulus” (GO: 0051606), and “cell killing” (GO: 0001906) were exclusively associated with root DEGs, thus highlighting the essential role of roots in plants’ responses to drought. Moreover, DEGs involved in “response to stimulus” (GO: 0006950) seem to play a pivotal role in drought sensing and the responses.

The GO enrichment analysis was performed using a *Q*-value < 0.05. The results revealed that, in the analysis of the root tissue, there were significant differences in the 26 GO terms between the DEGs and the genome reference, while only three molecular GO terms were enriched in the analysis of the leaves (“heme”, “iron”, and “tetrapyrrole binding” data not shown). In the analysis of the roots, the enriched GO terms were related to BP and MF (12 and 14 GO terms, respectively, as shown in Supplementary Figure S4). The most significant BP terms, such as “responses to stimulus” (GO: 0050896), “responses to stress” (GO: 0006950), and “biotic stimulus” (GO: 0009607), are illustrated in Supplementary Figure S5.

**FIGURE 4:**
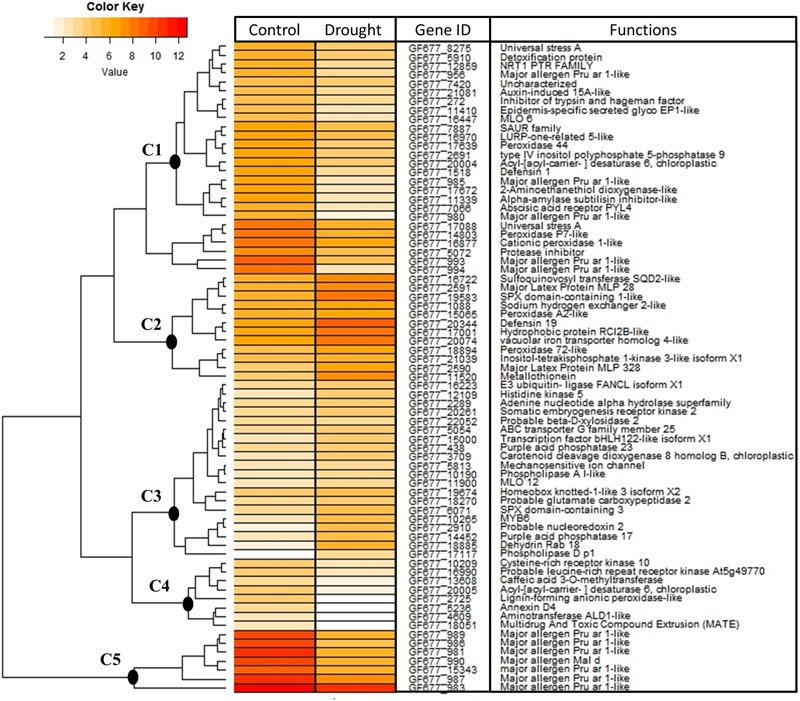
**Heat map of the DEGs in roots (GF677 rootstock) that are involved in “response to stimulus” (GO: 0050896).** The colors indicate the abundance of transcripts calculated as Log_2_ (RPKM+1) in the control and drought-stressed plants (see color key). The main gene clusters are numbered from C1 to C5. Further information about each gene is provided in Supplementary Table S2, listed, and grouped in clusters.

A heat map of DEGs involved in the “response to stimulus” is shown in **Figure 4** (see list in Supplementary Table S2). These genes were clustered into five clades according to their expression patterns. The genes in clusters C1, C4, and C5 had higher levels of expression in the control plants than in the drought-stressed plants; they mainly encode peroxidases and proteins related to “responses to biotic stimulus”, such as major allergen proteins, which might indicate that drought turns off this machinery. The remaining clusters (C2 and C3) comprised genes with higher levels of expression in the drought-stressed plants than in the control plants and they included kinases, transcription factors (TFs), and genes related to phosphate starvation.

### Classification of Drought-Inducible Genes in GF677 Rootstock Budded with the Catherina Cultivar

After annotation, the DEGs in both tissues were classified into two major categories: regulatory genes (genes implicated in signaling and transcriptional regulation) and functional genes (genes that encode proteins that are directly involved in cell protection and damage repair). These genes are described in the next sections and they are further detailed in Supplementary Tables S3A,B.

#### Expression of Drought Stress Regulatory Genes

**FIGURE 5:**
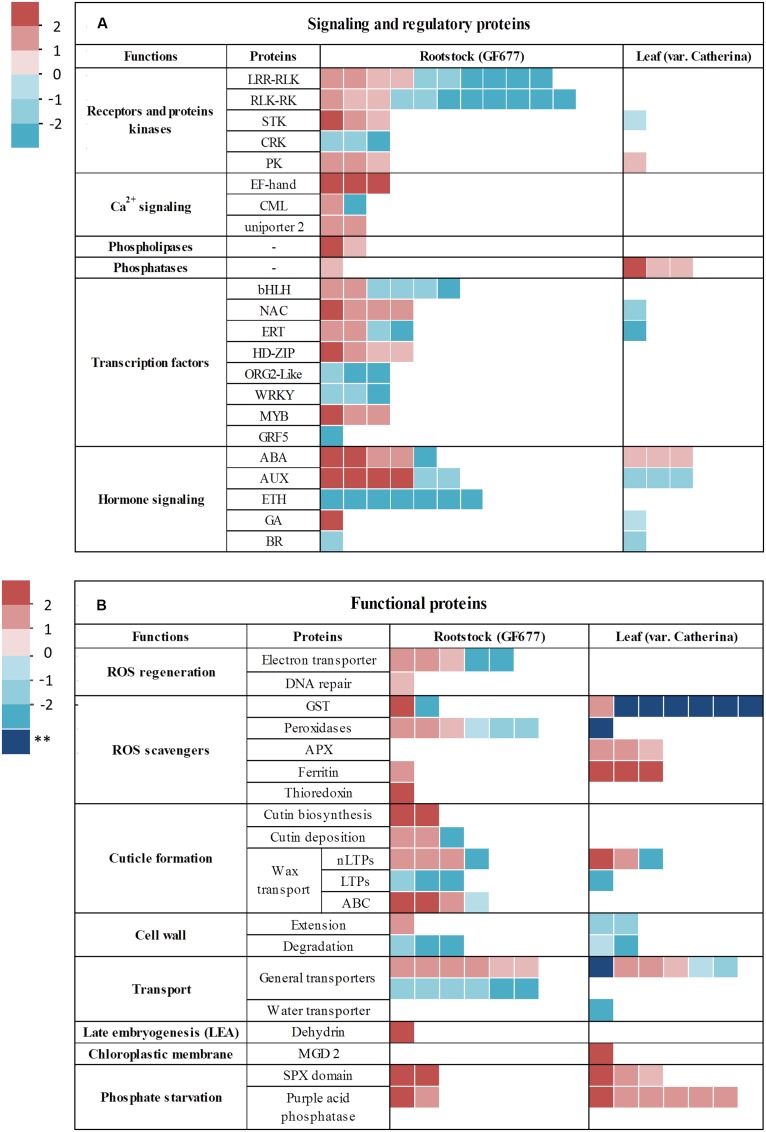
**Levels of expression of DEGs identified in roots (GF677 rootstock) and leaves (graft, var. Catherina). (A)** DEGs involved in signaling and regulatory processes. **(B)** DEGs involved in functional processes. Details are provided in Supplementary Table S3. The scale bar on the left represents the observed changes in expression in terms of Log_2_FC from upregulation (red squares) to downregulation (blue squares). The dark blue pattern with stars indicates genes uniquely expressed in leaves in the control group. The fold change was calculated as the ratio between the drought-stressed and control plants. ERT, ethylene-responsive transcription factor; GRF5, growth-regulating factor 5; ABA, abscisic acid; ETH, ethylene; AUX, auxin; GA, gibberellin; BR, brassinosteroid; APX, ascorbate peroxidase; nLTPs, non-specific-lipid transfer proteins; LTPs, lipid transfer proteins; ABC, ATP-binding cassette.

Regulatory genes play an important role in eliciting responses to abiotic stress. In this study, we detected 103 DEGs involved in signaling and regulation, of which only 15 were leaf DEGs (**Figure 5A**; Supplementary Table S3A). These DEGs included protein kinases and receptors (32), calcium sensors (7), phospholipases (2), phosphatases (4), TFs (30), and hormone-related genes (28). The identification of such a large number of genes indicates that plants use a large array of signaling mediators and complex pathways to combat drought stress.

Amongst the genes of the protein kinases, the receptor-like kinase (RLK) gene and the leucine-rich repeat receptor-like kinase (LRR-RLK) gene were the most redundant largely exhibited downregulation under conditions of drought. Likewise, the genes of the cysteine-rich receptor-like kinases (CRKs) exhibited downregulation. In contrast, the genes of kinases groups [serine-threonine kinases (STKs) and protein kinases (PKs)] were all upregulated with the exception of cvCatherina.11364. Regarding the phospholipases (Phospholipases A and D), they were upregulated exclusively in the roots.

A substantial number of the DEGs were TF genes, which were distributed into eight major families, based on their DNA-binding domains: bHLH (6), NAC (5), ERT (5), HD-ZIP (4), ORG2-like (3), WRKY (3), MYB (3), and growth-regulating factor 5 (GRF5) (1). As a result of the pivotal role of hormones as regulatory compounds, several of the DEGs were found to be hormone-related genes, which were related to the following hormones: abscisic acid (ABA), auxins, ethylene, gibberellins (GAs), and brassinosteroids (BRs); these hormones exhibited varying expression patterns. These results indicate that drought stress drives changes in the expression of many regulatory genes which serve as key components of signal transduction pathways.

#### Expression of Drought Stress Functional Genes

We identified 92 DEGs involved in functional processes, of which 39 were leaf DEGs (**Figure 5B**; Supplementary Table S3B). One of the inevitable consequences of drought stress is the enhanced production of reactive oxygen species (ROS). Five electron carriers were detected only in the roots, providing evidence about the initiation of redox signaling, which was also highlighted in the GO analysis. However, in addition to the role of ROS products as secondary messengers during drought, they can also induce oxidative damage. Plants have evolved several enzymatic compounds in order to maintain redox homeostasis. The DEGs were founds to encode three types of these enzymes: glutathione *S* transferases (GSTs, 9), peroxidases (7), and ascorbate peroxidases (APXs, 3), and non-enzymatic machinery including ferritins (4), and thioredoxin (1). As shown in **Figure 5B**, the non-enzymatic genes all exhibited upregulation while some enzymatic compounds were downregulated, and seven of the DEGs were expressed in the leaves only under control conditions.

In addition, DEGs involved in cuticle formation were found, including genes involved in cutin biosynthesis and deposition (5) and wax transport (15). These large numbers of DEGs imply that the cuticle may undergo extensive remodeling during drought as part of the plant’s adaptive survival mechanism (**Figure 5B**; Supplementary Table S3B). Furthermore, we identified several DEGs that were involved in cell wall extension (3) and degradation (5), which highlights the fact that plant cell walls constitute a major frontline of the plant defense system.

A total of 19 DEGs were annotated as transporters, including an aquaporin gene that was downregulated in the leaves (cvCatherina.12386, see Supplementary Table S3B). Late embryogenesis-abundant (LEA) genes are commonly induced during drought stress, thus we identified the LEA gene GF677_18885, which corresponds to the dehydrin Rab18. Based on the pivotal role of phosphorous in plant life, we identified 13 genes related to phosphate starvation, all of which were upregulated in both the roots and the leaves.

### KEGG Pathway Analysis

In order to look into the pathways that the DEGs were involved in, KEGG analysis was carried out. The major classes identified in roots and leaves are included in Supplementary Figure S6, which were Metabolism of amino acid in roots (31 DEGs, Supplementary Figure S6A) and Xenobiotics biodegradation and metabolism in both tissues (22 DEGs in the roots and 25 DEGs in the leaves). The complete set of matched pathways is summarized in Supplementary Table S4. Of the 500 DEGs in the roots, 152 had significant matches in the KEGG database (128 enzymes) and they were classified into 63 pathways (Supplementary Table S4A). In the leaves, 88 genes were assigned to 36 KEGG pathways and associated with 50 enzymes (Supplementary Table S4B). The major pathways identified in the roots were phenylpropanoid biosynthesis (nine DEGs and three enzymes) and aminobenzoate degradation (nine DEGs and four enzymes). In the leaves, glutathione metabolism (10 DEGs and four enzymes), aminobenzoate degradation (10 DEGs and two enzymes), and drug metabolism associated with cytochrome P450 (six DEGs and one enzyme) had the highest levels of differential expression.

### RT-qPCR Validation of DEGs from the RNA-seq Analysis

**FIGURE 6:**
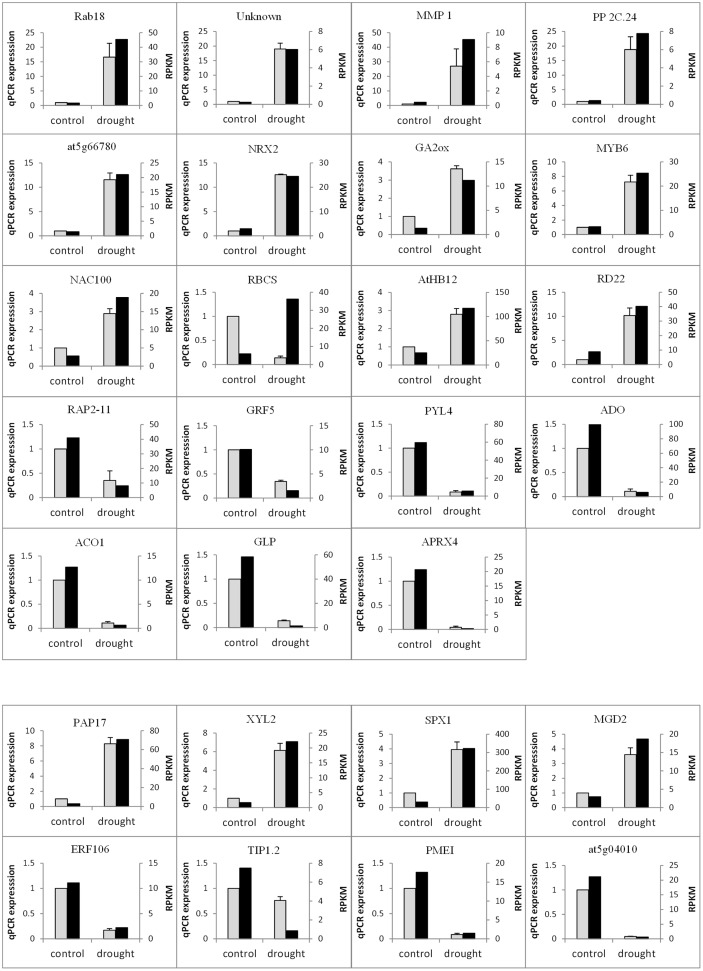
**Quantitative reverse transcription polymerase chain reaction (RT-qPCR) validation of selected genes in roots (GF677 rootstock) and leaves (graft, var. Catherina) in the control and drought-stressed plants.** The gray bars represent the relative expression determined by RT-qPCR (left *y*-axis) and the black bars represent the level of expression (RPKM) of the transcripts (right *y*-axis). The relative expression in the RT-qPCR analysis was normalized to the level in the GF677 rootstock of the control plants. The error bars indicate the standard error of quad-biological and bi-technical replicates. See abbreviations and further information about each gene in Supplementary Table S1.

**FIGURE 7:**
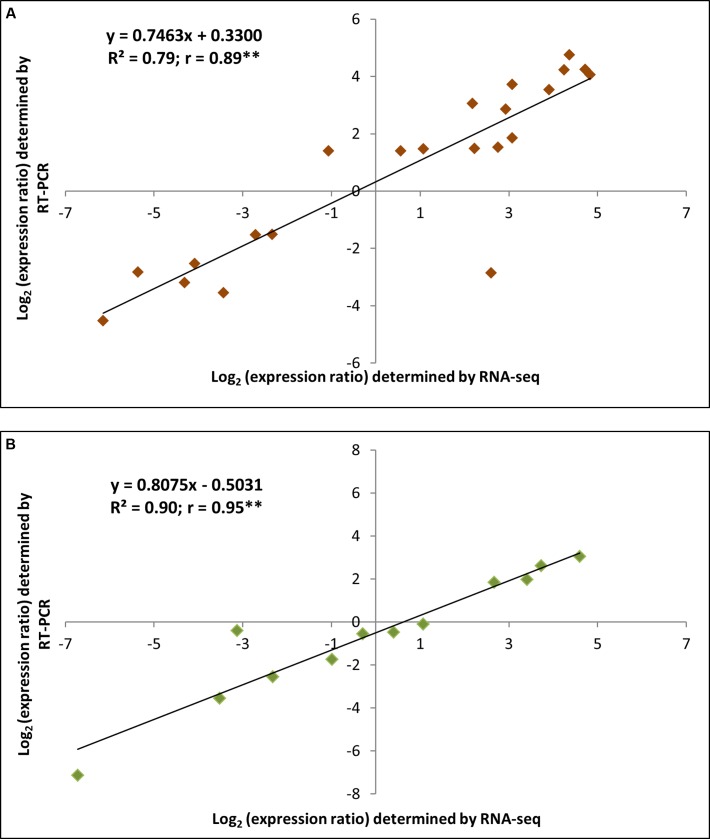
**Linear regressions involving the RNA sequencing data and the RT-qPCR validation data expressed in terms of Log_2_FC.** The fold change (FC) was calculated as the ratio between the drought-stressed and control plants. **(A,B)** indicate roots and leaves, respectively. ^∗∗^Significant Pearson’s correlation coefficient at *P* ≤ 0.01.

In order to further confirm the accuracy of the RNA-seq expression estimates, a total of 34 candidate genes were selected for RT-qPCR validation according to their RPKM transcript abundance and Log_2_FC. As illustrated in **Figure 6**, the expression values of the selected DEGs in both tissues significantly correlated with the RPKM values, with the exception of the chloroplastic ribulose bisphosphate carboxylase small chain (RBCS) gene, which may be a result of the unstable expression of this chloroplastic gene. The correlation between the RNA-seq and RT-qPCR measurements was evaluated using linear regression, based on the following equation: RT-qPCR value = b (RNA-Seq value) + a (**Figure 7**). Interestingly, the linear regression analysis indicated a highly significant correlation between the methods, indicating a general agreement regarding the transcript abundance determined by both methodologies (*r* = 0.89 and *r* = 0.95 for root and leaf DEGs, respectively). In conclusion, the obtained results confirm the reliability of the transcriptomic profiling data estimated from RNA-seq data.

## Discussion

### Physiological Responses to Drought

As sessile organisms, plants are unable to escape when environmental conditions become unfavorable. Nevertheless, they can successfully deploy complex physiological and molecular strategies to cope with environmental stresses. In this study, the physiological measurements confirmed that the plants were effectively subjected to a water deficit, and the plants elicited physiological responses to combat it. In fact, when water availability is limited, plants change their biochemistry in order to be able to retain as much water as possible and increase their chances of survival. One of the earliest responses to minimize water loss is the reduction of stomatal conductance, which leads to a reduction in CO_2_ diffusion through the stomata pores. The results concurred with the findings of several other studies that showed that water scarcity significantly reduces the rate of photosynthesis (Jiménez et al., 2013) by affecting the CO_2_ balance and stomatal status (Rahmati et al., 2015).

### Insight into the *Prunus* spp. Transcriptome

In order to investigate the dynamic changes in gene expression in the roots of GF677 rootstock and the leaves of Catherina cultivar budded together, RNA-seq was employed using the Illumina platform. Surprisingly, after quality control, the total number of clean reads generated from the RNA libraries of the drought-stressed plants was lower than the number for the control plants (**Table 2**). This is in contrast with previous studies, which have reported an activated plant transcriptome in response to drought (Tang et al., 2013; Garg et al., 2016). We thus hypothesize that a drought period of 16 days may be too short to drive the full upregulation of the *P. persica* genome, especially as the rootstock used was selected for its tolerance to drought (Jiménez et al., 2013).

The most frequent BLASTX top hits in our sequence homology searches were from *P. persica* and *P. mume*. These similarities highlight the quality of the assembly process. The annotation of the DEGs revealed a considerable number of hypothetical or uncharacterized protein functions, and some of these genes had large changes in expression. These genes could provide a good starting point for further experimental characterization. Generally, proteins with unknown functions are widespread across species, even in model plants. Indeed, in *Arabidopsis thaliana*, 13% of the genes encode proteins with unknown functions (Luhua et al., 2013). In spite of this, such genes are potentially interesting as they may encode proteins that would be valuable to breeders. Elucidating their biological roles in *Prunus* spp. is thus an important challenge, which we aim to achieve in future studies.

After GO annotation, the DEGs were labeled with 56 terms within three main categories (BP, MF, and CC). The most dominant terms, illustrated in Supplementary Figure S3, concur with the findings of previous research, confirming their universal involvement in the response to drought stress conditions (Dong et al., 2014; Li et al., 2016). In addition, some GO terms such as “locomotion”, “hormone metabolic process”, “detection of stimulus”, and “cell killing” were exclusively associated with root DEGs (**Figure 3**), indicating that despite being sessile, *P. persica* roots exhibit dynamic changes in architecture in response to water scarcity. An induced phospholipase D (PLD), associated with the regulation of cell migration and root development, is likely to be involved in locomotion. This observation likely reveals that, under conditions of drought stress, roots move downward in order to find water and escape from the harmful external factors. In fact, PLD hydrolyzes lipids, which results in the formation of phosphatidic acid (PA), a compound that is responsible of inducing cell proliferation and primary root growth (McLoughlin and Testerink, 2013). Functions related to the detection of stimuli were found to be increased in the roots during the drought responses of *P. persica*, confirming that these organs are responsible for sensing water deprivation. According to previous studies, the main steps for handling any type of abiotic stress are signal perception, signal transduction, and expression of stress-inducible genes. Thus, we propose that root cells first perceive drought through sensors located in the cell wall and/or membrane and then they convey the signals to other organs. Indeed, we have found that “membrane” and “cell” were the most dominant GO terms in the CC category, particularly regarding root DEGs. The “hormone metabolic processes” (GO: 0042445) associated with the root DEGs will be further discussed in Section “Signaling and Regulatory Proteins.”

### Generic Signaling Pathways Involved in *Prunus* spp. during Drought Stress

We observed that the initiation of drought stress triggered a wide range of responses, which implies that there are many genes and mechanisms involved in drought tolerance in *P. persica*. According to their associated proteins, we classified the DEGs as signaling and regulatory DEGs or functional DEGs.

#### Signaling and Regulatory Proteins

##### Receptor kinases

Stress perception is the first step involved in the activation of adaptive responses to ensure plant survival. The detection of extracellular stress signals is generally carried out via the receptor kinases on the cell walls and membranes, which bridge the gap between the perception of stress and signal transmission to the target genes. The majority of differentially expressed receptor kinases were found in the roots, supporting the notion that these organs are the primary sensors of drought stress. RLKs, including LRR-RLKs, formed the largest gene family in the data. However, most of them were strongly downregulated, whereas the remainder were slightly upregulated (**Figure 5A**; Supplementary Table S3A). It is well-documented that LRR-RLKs play roles in both biotic and abiotic stress responses (Osakabe et al., 2014); thus, we hypothesize that drought stress downregulates the biotic-response machinery, as shown in **Figure 4** (see, for instance, C5). This negative feedback could potentially be due to the repression of some LRR-RLKs, which was also observed for CRKs.

##### Ca^2+^ signaling

Following signal perception, the signals are relayed to downstream secondary messenger molecules, which are mainly calcium ions (Ca^2+^), ROS, and phytohormones, in order to initiate the signal transduction pathway. Ca^2+^ serves as versatile signaling messenger in response to various abiotic stimuli. The cytosolic concentration of Ca^2+^ has been found to increase due to the activation of Ca^2+^ channels during drought and salinity stress (Knight et al., 1997). Perturbations in the cytosolic concentration of Ca^2+^ are recognized by calcium-binding proteins (CBPs) that function as Ca^2+^ sensors, of which EF-hand CBPs are the major type (Batistič and Kudla, 2012). The DEGs involved in Ca^2+^ signaling were strongly induced in the roots except for GF677_3137 (Supplementary Table S3A). The upregulation of calcium uniporters, which transport Ca^2+^ from the cytosol to the mitochondrial matrix, suggests that drought stress could increase the Ca^2+^ concentration in *Prunus* spp. as way of maintaining the structural rigidity of the cell wall, which is in agreement with the results of previously cited studies (Knight et al., 1997; Batistič and Kudla, 2012). Furthermore, the upregulation of CBPs indicates that there is an enhancement of the intracellular signal transduction in *Prunus* spp. roots that are exposed to drought. The results may imply that EF-binding CBPs have a key role in sensing the Ca^2+^ signals and relaying the information to the rest of plant regulatory system.

##### Protein kinases and phospholipases

In contrast to the CBPs, PKs are sensor responders (Batistič and Kudla, 2012) that initiate phosphorylation cascades and thereby play important roles in responses to drought stress (Singh and Laxmi, 2015). The genes of STKs and PKs were upregulated which indicates the initiation of phosphorylation cascades. A notable DEG that was upregulated during conditions of drought was the inositol-tetrakisphosphate 1-kinase gene (GF677_21039, see Supplementary Table S3A). This member of the inositol pyrophosphate (IP) family has been reported to catalyze the production of inositol 1,3,4,5,6 pentakisphosphate IP_5_, which acts as a secondary messenger during environmental stress (Worley et al., 2013). On the other hand, we identified induced genes that encoded diacylglycerol kinase (DKG, cvCatherina.7529) and phospholipase D (PLD, GF677_17117), which are considered to be key generators of PA, a major root lipid signaling molecule during conditions of drought (Arisz et al., 2009; Hong et al., 2010; McLoughlin and Testerink, 2013).

##### Transcription factors

At the end of signaling cascades, TFs, broadly categorized as early-induced genes, are targeted by PKs and phosphatases (Hussain Wani et al., 2016). In the present study, drought significantly influenced transcription regulation, especially in the roots (in contrast, only two leaf TFs were annotated). This suggests that the transcriptional reprogramming of stress-responsive genes is initiated in the roots, reflecting their pivotal regulatory role.

Amongst the genes of the TFs, the bHLH genes were the most redundant largely exhibited downregulation under conditions of drought. However, bHLH122 was found to be induced, which concurs with its previously reported role in drought tolerance in *A. thaliana*, where it increases levels of cellular ABA by repressing the catabolism of ABA (Liu et al., 2014). NAC factors are known to play diverse roles in stress responses (Bianchi et al., 2015). In particular, GF677_17765 may encode NAC29, which was found to delay senescence and boost primary root elongation in transgenic *A. thaliana* roots (Huang et al., 2015). Similarly, HD-ZIP and MYB TFs were strongly induced during drought, shedding light on their putative roles as mediators of drought signaling (Chew et al., 2013; Chen et al., 2014). In particular, the gene encoding transcript GF677_6534 is a putative ortholog of the ABA-dependent AtHB12 (a HD-Zip gene, which was validated by RT-qPCR) found in *A. thaliana*, which promotes root elongation during mild drought stress (Ré et al., 2014). Thus, after considering the previously discussed “locomotion” annotation (**Figure 3**), we hypothesize that GF677_6534 may control primary root elongation during conditions of drought. This would contribute to the annotation of members of the HD-Zip family in *Prunus* spp., of which only the AtHB8 gene has been functionally characterized (Zhang et al., 2014). The repression of WRKY70 is in agreement with previous reports that have highlighted its role as a negative regulator of cell senescence (Griffiths et al., 2014). Interestingly, among the TFs, we also found that GRF5 was repressed in the roots and its expression was validated by RT-qPCR. The family of GRFs comprises 10 TFs in *P. persica*, but the description of their functions is still incomplete (see annotations, for instance at http://planttfdb.cbi.pku.edu.cn/search.php). According to studies on *A. thaliana*, GRF is involved in leaf and root expansion (Omidbakhshfard et al., 2015), although its regulatory effect over pivotal and lateral roots remains unclear.

##### Hormone signaling (Phytohormones)

One of the major signaling molecules used during drought is ABA. The key step in its synthesis is catalyzed by 9-*cis-*epoxycarotenoid dioxygenase (NCED3). The transcript GF677_11309 encodes NCED3 and it was significantly induced in roots, confirming that a water deficit enhances the synthesis of ABA (Supplementary Table S3). In agreement with previous reports, protein phosphatases PP2C, which are major ABA regulators, were also upregulated (Tang et al., 2013; Yang et al., 2015; Iovieno et al., 2016; Magalhães et al., 2016). The accumulation of ABA leads to activation of ABA-dependent TFs, such as MYB factors, as was shown in this study. For instance, the promoter of the drought-inducible gene RD22 (GF677_3749, Supplementary Table S1), which was validated by RT-qPCR, is known to harbor *cis*-elements that can be bound by MYB TFs in *A. thaliana* (Yamaguchi-Shinozaki and Shinozaki, 1993). Furthermore, in the roots we found three indole-3-acetic-acid-amido synthetases with Log_2_FC > 2 that adjust cell auxin levels via the inactivation of indole-3-acetic acid (IAA), one of the major forms of auxins in plants (Böttcher et al., 2012). The upregulation of these genes co-occurred with the downregulation of auxin-responsive proteins (auxin-binding protein family and the SAUR family) (Supplementary Table S3A). Indeed it is well-documented that auxin is key plant hormone that promotes lateral root formation (Lavenus et al., 2013). Thus, we propose that the inhibition of IAA is an adaptive survival strategy to reduce lateral roots emergence as their maintenance requires metabolic investment that may slow the axial root elongation into deep soil. This is important for the acquisition of water, which availability is higher in deep soils.

Seven root DEGs encoding aminocyclopropane-1-carboxylate oxidase (ACO1), a key enzyme in the ethylene biosynthesis pathway, were strongly downregulated. Likewise, most of the ethylene-responsive transcription factors (ERTs) followed the same pattern, as they are sensors of ethylene (Müller and Munné-Bosch, 2015). As ethylene has been linked to the promotion of cell senescence (Griffiths et al., 2014), we propose that the inhibition of ethylene biosynthesis is a mechanism to reduce the effects of drought, probably in coordination with NAC29, which also delays senescence (Huang et al., 2015).

GA2ox (GF677_10709, Supplementary Table S3A; **Figure 5A**) controls the endogenous level of Gibberellin (GA) by deactivating bioactive GA (Zawaski and Busov, 2014). The overexpression of GA2ox leads to GA deficiency in the plant, which maybe a mechanism to confer drought tolerance in *P. persica.*

The data indicate that there were two repressed BR-responsive genes in both tissues, which supports the findings of molecular studies that have reported that there is crosstalk between BR and other hormones (GA, auxin, and ethylene) (Bajguz and Hayat, 2009). Overall, our results indicate the prominent role of ABA-regulated responses to drought, while the all other major hormones and related pathways are generally downregulated.

#### Functional Proteins

Electron transporters are the major site of ROS production (Cruz de Carvalho, 2008). Upregulation of the DEGs associated with electron transporters increases the electron flux, thereby increasing ROS production and disturbing the ROS balance. Overproduction of ROS is extremely harmful to plants as it causes lipid oxidation, DNA damage, and programmed cell death (Das and Roychoudhury, 2014). Interestingly, in the roots, we identified an induced E3 ubiquitin-protein ligase FANCL (previously described in studies on humans), which is involved in DNA repair (GF677_16223, Supplementary Table S3B). Based on this, we suggest that this protein may repair oxidative DNA damage. Furthermore, ROS scavengers were mainly expressed in the leaves, which confirm that this tissue is more susceptible to oxidative damage than root tissue (see in Supplementary Figure S6B that Ascorbate and aldarate metabolism is upregulated in the leaves). Indeed, it has been documented that ROS generation mainly occurs in photosynthetic tissues, in chloroplasts and mitochondria (Das and Roychoudhury, 2014). Moreover, non-enzymatic scavenging genes were upregulated, highlighting the need to protect against oxidative stress. Although, studies have reported high levels of GST activity during drought (Liu et al., 2015; Garg et al., 2016), six of the genes associated with GST were exclusively expressed in the leaves of the control plants and they were not detected in the drought-stressed plants. These may be due to the effect of the drought-tolerant GF677 rootstock masking the effects of the drought. The fact that GSTs serve as auxin-binding proteins (Marrs, 1996) may also explain our findings as this hormone was found to be downregulated in both tissues.

The role of detoxification enzymes in cell protection has been well-documented in other plants (Sappl et al., 2009; Das and Roychoudhury, 2014), as well as the roles of proteins such as nucleoredoxin, multidomain thioredoxin, and ferritin (Kang and Udvardi, 2014; Li and Wei, 2016). In this study ferritins were found to be upregulated in the drought-stressed plants, potentially in order to sequester free iron that would otherwise catalyze the Fenton reaction and produce highly reactive hydroxyl radicals.

The cuticle is composed of two layers, an inner layer of cutin and an layer of outer wax, to ensure that the plant has hydrophobic protection against water loss (Yeats and Rose, 2013). Genes involved in cutin biosynthesis and deposition were expressed at high levels in the roots of drought-stressed plants. This may help to reinforce plants’ first-line barrier as drought can weaken roots, making them more susceptible to biotic attack. DEGs encoding wax transporters showed variable expression patterns: while lipid transfer proteins (LTPs) were repressed, non-specific lipid transfer proteins (nLTPs) and ABC (ATP-binding cassette) transporters were mostly activated. Based on these findings, we suggest that although drought negatively affected LTPs, the other transporters may play an important role in wax accumulation, and thus enhance the rigidity of the plant, as reported in coffee plants undergoing drought stress (Mofatto et al., 2016).

Plants experiencing low water availability face the challenge of reducing their leaf area. Expansins play a role in this process, by extending the cell walls, and they are known to be regulated by auxin (Perrot-Rechenmann, 2010). The results showed that there were two repressed expansins in the leaves. These observations suggest that leaves respond to reduce their levels of water loss. In addition, DEGs encoding enzymes involved in cell wall degradation were strongly downregulated in both tissues, which appears to be an adaptive way to increase cell wall rigidity. Transporter genes showed changes in expression in both directions. The most interesting example is perhaps the aquaporin TIP1.2, which was found to be downregulated in the leaves (Log_2_FC = -3.13, see Supplementary Table S3B). This expression pattern in the leaves is consistent with the measured levels of stomatal conductance (see **Table 1**), as suggested by Pou et al. (2013). In contrast, the dehydrin Rab18, an LEA protein, was significantly upregulated in the roots, which concurs with its expected role in protecting CCs from dehydration (Graether and Boddington, 2014).

Regarding the integrity of the chloroplasts, we found that monogalactosyldiacylglycerol synthase (MGD2) was upregulated in the leaves; this molecule is known to be involved in the biosynthesis of galactolipid, a molecule that stabilizes the chloroplast membrane, thereby ensuring the photosynthesis can be maintained (Wang et al., 2014).

Finally, transcripts encoding proteins that are involved in phosphate starvation were overexpressed in both tissues, which highlights the role of phosphate in many vital pathways, in particular, photosynthesis, signaling, and growth (Dos Santos et al., 2006).

## Conclusion

Drought tolerance is a complex trait that is controlled by multiple genes, and the identification of drought-inducible genes in this study provides an insight into the major mechanisms adopted by *P. persica* to tolerate periods of drought. This study represents an exploration of the global biological, molecular, and cellular responses of *P. persica* to stress using next-generation sequencing and computational methods. The results shed light on the role of ABA as the major drought-induced hormone, while the other hormones and related pathways were generally shown to be downregulated. Additionally, many DEGs that are associated with important pathways were identified, some of which are targets for further genetic studies of *P. persica*. In particular, two drought-induced genes, GRF5 and AtHB12, represent a starting point for investigations of poorly characterized genes in *P. persica*. However, when assessing the genes that are potentially involved in drought responses, it should be taken into account that plant responses depend largely on the severity and duration of the water deficit scenario.

## Author Contributions

SJ and YG devised the study objectives and designed the experiments. SJ and NK performed the research. NK and BC-M analyzed the data. YG, CW, and BC-M contributed the reagents, and the analysis tools. NK, BC-M, and YG discussed the data and wrote the manuscript. All the authors read and approved the final manuscript.

## Conflict of Interest Statement

The authors declare that the research was conducted in the absence of any commercial or financial relationships that could be construed as a potential conflict of interest.

## Footnotes

1 http://faostat.fao.org

2 www.ncbi.nlm.nih.gov/

3 https://phytozome.jgi.doe.gov/pz/portal.html

4 https://www.rosaceae.org/

5 http://wego.genomics.org.cn/cgi-bin/wego/index.pl

6 http://bioinfo.cau.edu.cn/agriGO/

7 http://www.ncbi.nlm.nih.gov/tools/primer-blast/
