# Supplementary material for: Transcriptional Responses in Root and Leaf of Prunus persica under Drought Stress Using RNA Sequencing
Source: Front Plant Sci. 2016 Nov 23;7:1715. doi: 10.3389/fpls.2016.01715 (PMC5120087; doi:10.3389/fpls.2016.01715)
Supplement: Supplementary file 1 [file Table_1.PDF]

Table S1. Primer list and amplicon sizes of differentially expressed genes (DEGs) in roots (GF677 rootstock) and leaves (graft, var. Catherina) selected for RT-qPCR validation. The gene ID is the same as that deposited in the ENA. The RPKM values are indicated for the control and drought-stressed plants. The fold change (FC) was calculated as the ratio between the drought-stressed and control plants. The grey rows indicate genes with $|Log_2FC| < 2$. GDR: Genome Database of Rosaceae.

Genes differentially expressed in roots

Gene ID	ID in GDR database	Locus	Description	Abbreviations	RPKM		Log_2FC	Forward	Reverse	Amplicon size
					Control	Drought				
**GF677_18885**	ppa011637m	scaffold_7:17140290-17142422	Dehydrin rab 18	Rab18	1.59	45.34	4.83	ATCGGTGCTTGTTGTTGGTC	TACTTGAAAGCAGCAGACAATAAA	151
**GF677_10293**	ppa009829m	scaffold_3:21224913-21415329	Unknown protein	Unknown	0.23	6.03	4.72	ATGGACGCTGGAGATGGTTG	GGCGAAATCGGACAGGAAGA	134
**GF677_9678**	ppa016355m	scaffold_3:20151304-20170331	Metalloendoproteinase 1-like	MMP1	0.44	9.05	4.36	TCCTAGCGCATTCTTTCGC	ATGCAAAGCCACAGTCTCCA	113
**GF677_15114**	ppa006696m	scaffold_6:4698391-4700540	Phosphatase 2C 24	PP2C.24	0.41	7.78	4.25	CCTGCACTACGTCGGTTTCT	AATCTTCAGGCTCGCAACGA	92
**GF677_18824**	ppa013228m	scaffold_7:16790537-16791344	at5g66780 mud21_2	at5g66780	1.40	21.05	3.91	TCCAGCGAGTCCGATAGTGA	CACTCCGAACCCATCGTCAT	138
**GF677_2910**	ppa006064m	scaffold_1:32257654-32261879	Probable nucleoredoxin 2	NRX2	2.90	24.53	3.08	ACTGCGGTACTCTTCTGTGC	CTCTGGAGCTTGAGACGGTG	140
**GF677_10709**	ppa008211m	scaffold_4:3917370-3919509	Gibberellin2 beta dioxygenase 2	GA2OX2	1.32	11.19	3.08	GCACCTCTGCCGTCACTTAT	TTTCTCGAACAGCCCTAGCC	126
**GF677_10265**	ppa007438m	scaffold_4:1665468-1667309	Transcription repressor MYB6	MYB6	3.32	25.32	2.93	CCGGAAGAACCGACAACGAA	AAGAAGGTCAAGACGAGGGC	110
**GF677_9569**	ppa007883m	scaffold_3:19548129-19549934	NAC domain containing protein100-like	NAC100	2.79	18.90	2.76	GACAGCTTCCAACACAACAAC	AAGGGTTTAAGTGAGCAGAGG	103
**GF677_8702**	ppa012123m	scaffold_3:12230346-12231657	Ribulose bisphosphate carboxylase small chain chloroplastic	RBCS	5.95	36.21	2.61	ACCTTCCACCACTCTCTCCT	CGGTACACAAATCCCTTCTCCA	109
**GF677_6534**	ppa010647m	scaffold_2:19683395-19685063	Homeobox- leucine zipper AtHB12-like	AtHB12	25.00	117.06	2.23	ACCCGAACAACCATCACCAA	CATCTTCTGCTGTGGGGCTA	121
**GF677_3749**	ppa005699m	scaffold_1:37149693-37151803	Dehydration-responsive protein RD22	RD22	8.89	40.23	2.18	CTTGCTTGCTTCCGTTCATC	TCCAGTAAAGCTGAGGTGGT	149
**GF677_10556**	ppa002450m	scaffold_4:3002171-3008328	LRR receptor-like serine/threonine-protein kinase At5g45840	LRR	4.21	8.89	1.08	GCTGCATAGCCAGGTGGACA	TGGTAGGTTACTGGCTTGTGC	106
**GF677_18270**	ppa026745m	scaffold_7:12589869-12593343	Probable glutamate carboxypeptidase 2	GCPII	10.21	15.11	0.57	TGTCCGCGCATTTTAGCAAC	AGGGAGAGTAGGCGTGGTAG	105
**GF677_721**	ppa008797m	scaffold_1:7495645-7497762	Cinnamoyl-CoA reductase 2-like	CCR2	30.51	14.60	-1.06	ACCGAAAGAGCGTGAATGGA	CTGTTTCCCCTGCATCAAACC	154
**GF677_17720**	ppa016109m	scaffold_7:4278166-4279839	Ethylene-responsive transcription factor RAP2-11	RAP2-11	40.94	8.14	-2.33	TTGTTGGTGTGAGGCAAAGG	TCGGCAGTCTCAAAGGTTCC	100
**GF677_7962**	ppa017593m	scaffold_3:2115917-2118205	Growth-regulating factor5-like	GRF5	10.09	1.54	-2.71	AGCCTGCAACCCACTATACA	AGAAGGATTCAGGAAGAACAGC	95
**GF677_7066**	ppa025240m	scaffold_2:23277709-23279257	Abscisic acid receptor PYL4-like	PYL4	59.46	5.52	-3.43	TGGAGTCATACGTGGTGGAC	ACTCTTGGTGGATGGTTTGG	137
**GF677_17672**	ppa009537m	scaffold_7:3370462-3373793	2-Aminoethanethiol Dioxygenase	ADO	100.52	5.94	-4.08	AATGGAGACTGCGAGGTTGG	TGGCACCGAATGCTTCATCT	142
**GF677_300**	ppa023251m	scaffold_1:2553496-2555109	1-aminocyclopropane-1-carboxylate oxidase homolog 1-like	ACO1	12.70	0.64	-4.31	ACTCGTAAAAGGGAACTCAGCA	AGAGTGACCACCAACCAACA	133
**GF677_14474**	ppa016616m	scaffold_5:18137563-18138859	Germin-like	GLP	58.36	1.42	-5.36	TGGGACACGTCAACGCTTTA	GTCGTCGATCTGGAATGCCT	137
**GF677_2725**	ppa023604m	scaffold_1:31141962-31143686	Lignin-forming anionic peroxidase 4-like	APRX4	20.68	0.29	-6.15	TCAGTTGCTGTGAGTGGTCC	AAATCAGGCGGTCAAGGGAG	124

Genes differentially expressed in leaves

Gene ID	ID in GDR database	Locus	Description	Abbreviations	RPKM		Log_2FC	Forward	Reverse	Amplicon size
					Control	Drought				
**cvCatherina.11767**	ppa008418m	scaffold_5:17984196-17986682	Purple acid phosphatase 17	PAP17	2.94	70.81	4.59	GGATGTCGAGTTTGCACTGA	ATTGGCCTGAAGGATTGGGA	136
**cvCatherina.14437**	ppa001232m	scaffold_7:6344692-6348608	Alpha-xylosidase 2	XYL2	1.67	22.09	3.73	GGTCTCCCAATGGTCTCAGT	ACTTGCAGTTTCCCATTTTCTG	128
**cvCatherina.15841**	ppa009473m	scaffold_7:21242038-21244975	SPX domain-containing 1-like	SPX1	30.42	321.97	3.40	AGCAGCCAGATCGAGGAAAC	ACAATCAGCGGAGGCATCAA	144
**cvCatherina.15894**	ppa006453m	scaffold_7:21656515-21659600	Monogalactosyldiacylglycerol synthase 2, chloroplastic	MGD2	2.95	18.68	2.66	GGGAGGTGGTGAAGGAATGG	TCTGGAGTGTAGAGGCGAGG	143
**cvCatherina.11558**	ppa006823m	scaffold_5:16453361-16456966	Glycerophosphoryl diester phosphodiesterase 3	SHV3	52.65	110.59	1.07	GTGGGCAGATGGCAAGAGAT	AGCGAAGTTGGAGCGAATGA	132
**cvCatherina.6050**	ppa011927m	scaffold_2:25645720-25648634	Abscisic acid receptor PYL8	PYL8	38.04	50.06	0.40	CGCAGACGGAAAACGAAAGG	GTCGTGCTTGTGATGCCTTC	155
**cvCatherina.13766**	ppa011255m	scaffold_6:25840498-25843531	Pyroglutamyl-peptidase 1-like	PGPEP1	82.33	67.07	-0.30	CCCTCCACTAACACGTTCCT	TCACAAGCCTCAACCACCAG	157
**cvCatherina.370**	ppa005761m	scaffold_1:4287856-4293128	Pectate lyase 1 related	PEL1	14.26	7.17	-0.99	GTGCTTCTCGTGCTTTGCTT	CTCATCATCCTCAGCCACCC	126
**cvCatherina.5807**	ppa022802m	scaffold_2:23997510-23998782	Ethylene-responsive transcription factor ERF106	ERF106	11.10	2.22	-2.32	AACCATAATCTCGCCATCCA	ACTTCACCAGAACCCGACAC	138
**cvCatherina.12386**	ppa010367m	scaffold_6:5724911-5726097	Aquaporin TIP1.2	TIP1.2	7.48	0.86	-3.13	ACAGTTTATGCCACAGCCCT	AAGGCACCAGCAACCAAGAT	107
**cvCatherina.5855**	ppa011607m	scaffold_2:24339289-24340181	Pectin methylesterase inhibitor	PMEI	17.60	1.53	-3.53	TGTCAAAGCAGCACTAACCCA	TTGGTGCCATGAGAGACTGC	146
**cvCatherina.7039**	ppa025502m	scaffold_3:11265705-11266708	Probable FBOX protein at5g04010	at5g04010	21.19	0.20	-6.72	AAATCCGCCACAATCCCACT	TGGAACGTGGTGTTGGAAGG	158

Reference genes

Gene ID	ID in GDR database	Locus	Description					Forward	Reverse	Amplicon size
__	ppa010708m	scaffold_2:9,706,751..9,710,146	AGL-26 LIKE					TGCAACAGTGAAACATTTGG	CATACAAAGCAATGCCAACA	103
__	ppa007238m	scaffold_5:12,941,307..12,944,365	Actin 2					ACTGGGACGACATGGAAAAG	GATTCAGGGGTGCCTCAGTA	102
