# Supplementary material for: Transcriptional Responses in Root and Leaf of Prunus persica under Drought Stress Using RNA Sequencing
Source: Front Plant Sci. 2016 Nov 23;7:1715. doi: 10.3389/fpls.2016.01715 (PMC5120087; doi:10.3389/fpls.2016.01715)
Supplement: Supplementary file 2 [file Table_2.PDF]

Table S2. Differentially expressed genes (DEGs) in roots (GF677 rootstock) involved in “response to stimulus” (GO: 0050896), with their homology-based predicted functions, RPKM in both conditions, and Log_2FC. The fold change (FC) was calculated as the ratio between the drought-stressed and control plants. The gene ID is the same as that deposited in the ENA.

**Clusters**	**Gene _ID**	**Functions**	**RPKM control**	**RPKM drought**	**Log_2 FC**	**Regulation**
**C1**	GF677_8275	Universal stress A	59.76	16.40	-1.87	Down
	GF677_5910	Detoxification protein	44.59	17.45	-1.35	Down
	GF677_12859	NRT1 PTR FAMILY	54.17	11.15	-2.28	Down
	GF677_956	Major allergen Pru ar 1-like	49.64	9.34	-2.41	Down
	GF677_7420	Uncharacterized	29.97	13.13	-1.19	Down
	GF677_21081	Auxin-induced 15A-like	33.26	9.89	-1.75	Down
	GF677_272	Inhibitor of trypsin and hageman factor	38.20	6.96	-2.46	Down
	GF677_11410	Epidermis-specific secreted glyco EP1-like	34.71	5.96	-2.54	Down
	GF677_16447	MLO 6	27.10	5.54	-2.29	Down
	GF677_7887	SAUR family	94.81	24.76	-1.94	Down
	GF677_16970	LURP-one-related 5-like	77.01	25.33	-1.60	Down
	GF677_17639	Peroxidase 44	84.19	15.58	-2.43	Down
	GF677_2691	Type IV inositol polyphosphate 5-phosphatase 9	74.94	16.97	-2.14	Down
	GF677_20004	Acyl-[acyl-carrier-] desaturase 6, chloroplastic	105.81	12.82	-3.05	Down
	GF677_1518	Defensin 1	89.47	10.17	-3.14	Down
	GF677_985	Major allergen Pru ar 1-like	121.19	5.52	-4.46	Down
	GF677_17672	2-Aminoethanethiol dioxygenase-like	100.52	5.94	-4.08	Down
	GF677_11339	Alpha-amylase subtilisin inhibitor-like	70.79	5.83	-3.60	Down
	GF677_7066	Abscisic acid receptor PYL4	59.46	5.52	-3.43	Down
	GF677_980	Major allergen Pru ar 1-like	65.82	1.93	-5.09	Down
	GF677_17088	Universal stress A	283.06	46.96	-2.59	Down
	GF677_14803	Peroxidase P7-like	295.23	40.24	-2.88	Down
	GF677_16877	Cationic peroxidase 1-like	258.69	75.04	-1.79	Down
	GF677_5072	Protease inhibitor	173.14	29.25	-2.57	Down
	GF677_993	Major allergen Pru ar 1-like	455.64	21.44	-4.41	Down
	GF677_994	Major allergen Pru ar 1-like	283.21	6.09	-5.54	Down
**C2**	GF677_16722	Sulfoquinovosyl transferase SQD2-like	49.99	189.76	1.92	Up
	GF677_2591	Major Latex Protein MLP 28	54.09	174.13	1.69	Up
	GF677_19583	SPX domain-containing 1-like	50.44	239.40	2.25	Up
	GF677_1088	Sodium hydrogen exchanger 2-like	50.44	119.84	1.25	Up
	GF677_15065	Peroxidase A2-like	53.96	105.24	0.96	Up
	GF677_20344	Defensin 19	98.10	412.72	2.07	Up
	GF677_17001	Hydrophobic protein RCI2B-like	91.80	353.57	1.95	Up
	GF677_20074	vacuolar iron transporter homolog 4-like	84.76	266.96	1.66	Up
	GF677_18894	Peroxidase 72-like	26.43	71.05	1.43	Up
	GF677_21039	Inositol-tetrakisphosphate 1-kinase 3-like isoform X1	24.69	62.77	1.35	Up
	GF677_2590	Major Latex Protein MLP 328	18.21	71.77	1.98	Up
	GF677_11520	Metallothionein	13.28	173.29	3.71	Up
**C3**	GF677_16223	E3 ubiquitin- ligase FANCL isoform X1	7.05	14.68	1.06	Up
	GF677_12109	Histidine kinase 5	6.11	14.34	1.23	Up
	GF677_2289	Adenine nucleotide alpha hydrolase superfamily	7.81	12.90	0.72	Up
	GF677_20261	Somatic embryogenesis receptor kinase 2	7.24	12.11	0.74	Up
	GF677_22052	Probable beta-D-xylosidase 2	6.57	10.54	0.68	Up
	GF677_5054	ABC transporter G family member 25	6.38	17.00	1.41	Up
	GF677_15000	Transcription factor bHLH122-like isoform X1	5.83	17.41	1.58	Up
	GF677_438	Purple acid phosphatase 23	6.49	19.72	1.60	Up
	GF677_3709	Carotenoid cleavage dioxygenase 8 homolog B, chloroplastic	4.51	18.20	2.01	Up
	GF677_5813	Mechanosensitive ion channel	4.92	9.05	0.88	Up
	GF677_10190	Phospholipase A I-like	4.98	8.35	0.74	Up
	GF677_11900	MLO 12	3.62	10.47	1.53	Up
	GF677_19674	Homeobox knotted-1-like 3 isoform X2	14.57	21.85	0.59	Up
	GF677_18270	Probable glutamate carboxypeptidase 2	10.21	15.11	0.57	Up
	GF677_6071	SPX domain-containing 3	7.44	36.98	2.31	Up
	GF677_10265	MYB6	3.32	25.32	2.93	Up
	GF677_2910	Probable nucleoredoxin 2	2.90	24.53	3.08	Up
	GF677_14452	Purple acid phosphatase 17	4.12	31.65	2.94	Up
	GF677_18885	Dehydrin Rab 18	1.59	45.34	4.83	Up
	GF677_17117	Phospholipase D p1	1.06	6.67	2.66	Up
**C4**	GF677_10209	Cysteine-rich receptor kinase 10	15.39	3.84	-2.00	Down
	GF677_16990	Probable leucine-rich repeat receptor kinase At5g49770	12.97	3.27	-1.99	Down
	GF677_13608	Caffeic acid 3-O-methyltransferase	10.73	2.11	-2.35	Down
	GF677_20005	Acyl-[acyl-carrier-] desaturase 6, chloroplastic	13.77	1.33	-3.37	Down
	GF677_2725	Lignin-forming anionic peroxidase-like	20.68	0.29	-6.15	Down
	GF677_5236	Annexin D4	7.74	0.47	-4.04	Down
	GF677_4609	Aminotransferase ALD1-like	4.71	0.34	-3.81	Down
	GF677_18051	Multidrug And Toxic Compound Extrusion (MATE)	4.88	1.09	-2.16	Down
**C5**	GF677_989	Major allergen Pru ar 1-like	1,039.98	50.45	-4.37	Down
	GF677_986	Major allergen Pru ar 1-like	965.52	37.81	-4.67	Down
	GF677_981	Major allergen Pru ar 1-like	1,363.10	44.12	-4.95	Down
	GF677_990	Major allergen Mal d	757.25	50.08	-3.92	Down
	GF677_15343	Major allergen Pru ar 1-like	530.08	54.64	-3.28	Down
	GF677_987	Major allergen Pru ar 1-like	2,075.70	212.63	-3.29	Down
	GF677_983	Major allergen Pru ar 1-like	6,681.83	1,319.04	-2.34	Down
