# Supplementary material for: Transcriptional Responses in Root and Leaf of Prunus persica under Drought Stress Using RNA Sequencing
Source: Front Plant Sci. 2016 Nov 23;7:1715. doi: 10.3389/fpls.2016.01715 (PMC5120087; doi:10.3389/fpls.2016.01715)
Supplement: Supplementary file 3 [file Table_3.pdf]

Table S3. Functional classification of annotated differentially expressed genes (DEGs) in roots (GF677 rootstock) and leaves (graft, var. Catherina). The gene ID is the same as that deposited in the ENA. GDR: Genome Database of Rosaceae. The color bar indicates the transition of the expression pattern in terms of Log_2FC from upregulation (red squares) to downregulation (blue squares). The dark blue pattern with stars corresponds to genes uniquely expressed in the leaves of the control plants. The genes that were validated using RT-qPCR are in bold. The fold change (FC) was calculated as the ratio between the drought-stressed and control plants. (A) Signaling and regulatory proteins. (B) Functional proteins.

A- Signaling and regulatory proteins

Functions		Gene ID	ID in GDR	Annotation	Tissues	Log_2FC
Receptors and protein kinases	LRR-RLK receptor kinases	GF677_7143	ppa000889m	Probably inactive LRR protein kinase At3g28040	root	1,59
		**GF677_10556**	**ppa002450m**	**LRR receptor-like serine threonine protein-kinase At5g45840**	**root**	**1,08**
		GF677_20261	ppa002871m	Somatic embryogenesis receptor kinase 2	root	0,74
		GF677_19662	ppa000884m	LRR receptor kinase TDR	root	0,64
		GF677_11379	ppa003884m	Probable LRR receptor-like serine threonine- kinase At3g14840	root	-1,65
		GF677_16990	ppa000921m	Probable LRR receptor kinase At5g49770	root	-1,99
		GF677_11080	ppa017185m	Probable LRR receptor-like serine threonine- kinase At4g36180	root	-2,25
		GF677_11081	ppa017185m	Probable LRR receptor-like serine threonine- kinase At4g36180	root	-2,65
		GF677_5955	ppa020571m	Probable LRR receptor-like serine threonine- kinase At3g47570	root	-2,79
		GF677_11863	ppa017796m	Probable LRR receptor kinase At5g49770	root	-3,82
	RLK and RK receptor kinases	GF677_17252	ppa006797m	Receptor-like cytosolic serine threonine-protein kinase rbk2	root	1,19
		GF677_8772	ppa000942m	Receptor protein kinase TMK1	root	0,67
		GF677_12937	ppa006942m	Probable receptor-like protein kinase At5g47070	root	0,53
		GF677_5741	ppa003241m	Probable receptor kinase At1g67000	root	-1,11
		GF677_5727	ppa004124m	Probable receptor kinase At1g67000	root	-1,71
		GF677_3514	ppa027139m	Receptor kinase 1	root	-2,21
		GF677_15053	ppb015337m	G-type lectin S-receptor-like serine threonine- kinase RLK1	root	-2,48
		GF677_15022	ppb016806m	G-type lectin S-receptor-like serine threonine- kinase RLK1	root	-2,72
		GF677_8917	ppa019342m	Wall-associated receptor kinase-like 1	root	-3,06
		GF677_8592	ppa018157m	Probable receptor kinase At1g67000	root	-4,56
		GF677_3515	ppa027139m	Receptor kinase 1	root	-4,79
	STK	GF677_19962	ppa023131m	Serine/threonine-protein kinase At5g01020-like	root	2,84
		GF677_8729	ppa004124m	Serine threonine- kinase CDL1	root	0,96
		GF677_19063	ppa007710m	Probable serine threonine- kinase Cx32, chloroplastic	root	0,79
		cvCatherina.11364	ppa007019m	Serine threonine- kinase HT1-like	leaf	-0,52
	CRK	GF677_10211	ppa002309m	Cysteine-rich receptor kinase 29	root	-1,46
		GF677_10210	ppa002615m	Cysteine-rich receptor kinase 15	root	-1,81
		GF677_10209	ppa022109m	Cysteine-rich receptor kinase 10	root	-2,00
	Protein kinases (PK)	GF677_21039	ppa021570m	Inositol-tetrakisphosphate 1-kinase 3-like isoform X1	root	1,35
		GF677_12109	ppa000361m	Histidine kinase 5	root	1,23
		GF677_9801	ppa002420m	Kinase PVPK-1	root	0,80
		cvCatherina.7529	ppa004529m	Diacylglycerol kinase 5	leaf	0,50
Ca^{2+} signaling	EF-hand family	GF677_3581	ppa016083m	Calcium-binding EF-hand family	root	2,82
		GF677_3574	ppa026792m	Calcium-binding EF-hand family	root	2,78
		GF677_3573	ppa026792m	Calcium-binding EF-hand family	root	2,28
	Calcium-binding CML	GF677_3582	ppa013983m	Calcium-binding CML10	root	1,94
		GF677_3137	ppa021408m	Probable calcium-binding CML41	root	-2,50
	Uniporter	GF677_18778	ppa006923m	Calcium uniporter 2, mitochondrial	root	1,95
		GF677_18777	ppa006923m	Calcium uniporter 2, mitochondrial	root	1,90
Phospholipases	-	GF677_17117	ppa000572m	Phospholipase D p1	root	2,66
		GF677_10190	ppa000303m	Phospholipase A I-like	root	0,74
Phosphatases	-	GF677_16384	ppa011449m	Acylphosphatase	root	0,60
		cvCatherina.6526	ppa009904m	Inorganic pyrophosphatase 1-like	leaf	2,61
		cvCatherina.7411	ppa011399m	Probable tyrosine- phosphatase At1g05000	leaf	0,97
		cvCatherina.8503	ppa022650m	Probable 2-carboxy-D-arabinitol-1-phosphatase	leaf	0,32

Table S3 (A) Signaling and regulatory proteins (continued).

Functions		Gene ID	ID in GDR	Annotation	Tissues	Log_2FC
Transcription factors	bHLH	GF677_251	ppa017791m	Transcription factor bHLH92	root	1,80
		GF677_15000	ppa006295m	Transcription factor bHLH122-like isoform X1	root	1,58
		GF677_10526	ppa021837m	Transcription factor bHLH30-like	root	-1,11
		GF677_16183	ppa007656m	Transcription factor bHLH93	root	-1,55
		GF677_4929	ppa009461m	Transcription factor bHLH96-like	root	-1,65
		GF677_4782	ppa016514m	Transcription factor bHLH93-like	root	-2,31
	NAC	**GF677_9569**	**ppa007883m**	**NAC domain-containing 100-like**	**root**	**2,76**
		GF677_11557	ppa008301m	NAC domain-containing 72	root	1,98
		GF677_17765	ppa007445m	NAC transcription factor 29-like	root	1,87
		GF677_19364	ppa004775m	NAC domain containing 75 isoform 1	root	1,01
		cvCatherina.5318	ppa019780m	NAC domain-containing 72-like	leaf	-1,23
	ERT	GF677_16383	ppa003783m	AP2-like ethylene responsive transcription factor	root	1,25
		GF677_16245	ppa002612m	AP2-like ethylene-responsive transcription factor ANT	root	1,00
		GF677_21629	ppa023839m	Ethylene-responsive transcription factor 1B-like	root	-1,90
		**GF677_17720**	**ppa016109m**	**Ethylene-responsive transcription factor RAP2-11**	**root**	**-2,33**
		**cvCatherina.5807**	**ppa022802m**	**Ethylene-responsive transcription factor ERF106**	**leaf**	**-2,32**
	HD-ZIP	**GF677_6534**	**ppa010647m**	**Homeobox-leucine zipper AtHB12-like**	**root**	**2,23**
		GF677_18786	ppa011343m	Homeobox-leucine zipper protein athb-40	root	1,68
		GF677_3702	ppa001386m	Homeobox-leucine zipper AtHB-8	root	0,78
		GF677_19674	ppa007698m	Homeobox knotted-1-like 3 isoform X2	root	0,59
	ORG2	GF677_17863	ppa018670m	Transcription factor ORG2-like	root	-1,50
		GF677_17858	ppa024966m	Transcription factor ORG2-like	root	-2,15
		GF677_17857	ppa016095m	Transcription factor ORG2-like	root	-3,28
	WRKY	GF677_16920	ppa020736m	Probable WRKY transcription factor 70	root	-1,81
		GF677_16916	ppa020736m	Probable WRKY transcription factor 70	root	-1,93
		GF677_9903	ppa016459m	Probable WRKY transcription factor 75	root	-2,26
	MYB	**GF677_10265**	**ppa007438m**	**Transcription factor MYB 6**	**root**	**2,93**
		GF677_4292	ppa026006m	Transcription factor MYB39	root	1,97
		GF677_16023	ppa019923m	Transcription factor MYB39-like	root	1,53
	GRF	**GF677_7962**	**ppa017593m**	**Growth-regulating factor 5-like**	**root**	**-2,71**
Hormone signaling	ABA	**GF677_15114**	**ppa006696m**	**Probable phosphatase 2C 24**	**root**	**4,25**
		GF677_19254	ppa005286m	Phosphatase 2C 77	root	3,13
		GF677_11309	ppa002804m	9-cis-epoxycarotenoid dioxygenase NCED3	root	1,95
		GF677_19391	ppa006926m	Phosphatase 2C 56-like	root	1,85
		**GF677_7066**	**ppa025240m**	**Abscisic acid receptor PYL4-like**	**root**	**-3,43**
		cvCatherina.6471	ppa004252m	Phosphatase 2C 16-like	leaf	0,74
		cvCatherina.15676	ppa006926m	Phosphatase 2C 56-like	leaf	0,68
		**cvCatherina.6050**	**ppa011927m**	**Abscisic acid receptor PYL8**	**leaf**	**0,40**
	AUX	GF677_19295	ppa023151m	Auxin-induced in root cultures 12-like	root	2,93
		GF677_11622	ppa002986m	Indole-3-acetic acid-amido synthetase	root	2,76
		GF677_20924	ppa003134m	Probable indole-3-acetic acid-amido synthetase	root	2,71
		GF677_20923	ppa003134m	Probable indole-3-acetic acid-amido synthetase	root	2,01
		GF677_21081	ppa017462m	Auxin-induced 15A-like	root	-1,75
		GF677_7887	ppa013543m	SAUR family (Small auxin up RNA)	root	-1,94
		cvCatherina.6444	ppa011467m	Auxin-binding ABP19a	leaf	-1,65
		cvCatherina.6397	ppa013543m	SAUR family (Small auxin up RNA)	leaf	-1,81
		cvCatherina.6446	ppa011467m	Auxin-binding ABP19a	leaf	-1,98
	ETH	GF677_21642	ppa026451m	1-aminocyclopropane-1-carboxylate oxidase homolog 1-like	root	-2,14
		GF677_19946	ppa016953m	1-aminocyclopropane-1-carboxylate oxidase-like	root	-3,32
		GF677_19306	ppa009228m	1-aminocyclopropane-1-carboxylate oxidase 1	root	-3,92
		GF677_301	ppa022672m	1-aminocyclopropane-1-carboxylate oxidase homolog 1-like	root	-3,95
		GF677_302	ppa015518m	1-aminocyclopropane-1-carboxylate oxidase homolog 1-like	root	-4,22
		**GF677_300**	**ppa023251m**	**1-aminocyclopropane-1-carboxylate oxidase homolog 1-like**	**root**	**-4,31**
		GF677_21639	ppa022472m	1-aminocyclopropane-1-carboxylate oxidase homolog 1-like	root	-5,36
	GA	**GF677_10709**	**ppa008211m**	**Gibberellin 2-beta-dioxygenase-like**	**root**	**3,08**
		cvCatherina.2406	ppa013714m	Gibberellin-regulated 1-like	leaf	-0,95
	BR	GF677_8842	ppa020825m	Brassinosteroid-regulated protein BRU1-like	root	-1,20
		cvCatherina.4169	ppa009792m	Brassinosteroid-regulated BRU1-like	leaf	-1,55

Table S3 (B) Functional proteins.

B- Functional proteins

Functions			Gene ID	ID in GDR	Annotation	Tissues	Log$_2$FC	
ROS generation	Electron transporter		GF677_7191	ppa017806m	Mavicyanin-like	root	1,84	
			GF677_9227	ppa017076m	Blue copper protein	root	1,74	
			GF677_6176	ppa026142m	Cucumber peeling cupredoxin-like	root	0,73	
			GF677_19596	ppa009089m	Blue copper protein	root	-2,16	
			GF677_19598	ppa011524m	Blue copper protein like	root	-2,58	
	DNA repair		GF677_16223	ppa017844m	E3 ubiquitin- ligase FANCL isoform X1	root	1,06	
ROS scavengers	Glutathione S-transferases (GST)		GF677_11293	ppa011202m	Probable glutathione S-transferase	root	2,31	
			GF677_14079	ppa019399m	Glutathione S-transferase U10-like	root	-2,91	
			cvCatherina.9155	ppa011202m	Probable glutathione S-transferase	leaf	1,69	
			cvCatherina.13478	ppa018112m	Probable glutathione S-transferase	leaf	-1.80e+308	**
			cvCatherina.17367	ppa024281m	Probable glutathione S-transferase	leaf	-1.80e+308	**
			cvCatherina.1098	ppa022301m	Probable glutathione S-transferase	leaf	-1.80e+308	**
			cvCatherina.11474	ppa027220m	Glutathione S-transferase U9-like	leaf	-1.80e+308	**
			cvCatherina.351	ppa024247m	Probable glutathione S-transferase	leaf	-1.80e+308	**
			cvCatherina.266	ppa011087m	Probable glutathione S-transferase	leaf	-1.80e+308	**
	Peroxidases		GF677_20376	ppa023313m	Peroxidase 2	root	1,64	
			GF677_18894	ppa008349m	Peroxidase 72-like	root	1,43	
			GF677_15065	ppa007826m	Peroxidase A2-like	root	0,96	
			GF677_16877	ppa008642m	Cationic peroxidase 1-like	root	-1,79	
			GF677_17639	ppa009513m	Peroxidase 44	root	-2,43	
			GF677_14803	ppa018701m	Peroxidase P7-like	root	-2,88	
			cvCatherina.473	ppa008569m	Peroxidase 16	leaf	-1.80e+308	**
	Ascorbate peroxidase (APX)		cvCatherina.12438	ppa010431m	Cytosolic ascorbate peroxidase	leaf	1,36	
			cvCatherina.13321	ppa010426m	L-ascorbate peroxidase 2, cytosolic	leaf	1,22	
			cvCatherina.3325	ppa006270m	Probable L-ascorbate peroxidase 6, chloroplastic isoform X1	leaf	0,20	
	Ferritins		GF677_16815	ppa010086m	Ferritin-4	root	1,66	
			cvCatherina.5689	ppa009703m	Ferritin-3, chloroplastic-like	leaf	2,82	
			cvCatherina.13605	ppa010086m	Ferritin-4, chloroplastic-like	leaf	2,35	
			cvCatherina.13603	ppa008598m	Mitoferrin-like	leaf	2,27	
	Thioredoxin		**GF677_2910**	**ppa006064m**	**Probable nucleoredoxin 2**	**root**	**3,08**	
Cuticle formation	Cutin biosynthesis		GF677_7917	ppa004479m	3-oxoacyl-[acyl-carrier-protein] synthase II (KAS II)	root	2,54	
			GF677_223	ppa004243m	3-ketoacyl- synthase 11-like (KCS)	root	2,20	
	Cutin deposition		GF677_15537	ppa007967m	GDSL esterase lipase At5g22810	root	1,61	
			GF677_18716	ppa021185m	GDSL esterase lipase At2g23540	root	1,46	
			GF677_7885	ppa026827m	GDSL esterase lipase 2-like	root	-2,87	
	Wax transport	nLTPs	GF677_5697	ppa013464m	Non-specific lipid-transfer 8-like	root	1,84	
			GF677_21746	ppa021933m	Non-specific lipid-transfer At2g13820	root	1,63	
			GF677_21748	ppa011432m	Non-specific lipid-transfer At2g13820	root	1,48	
			GF677_14820	ppa013519m	Non-specific lipid-transfer 1-like	root	-4,95	
			cvCatherina.12046	ppa013519m	Non-specific lipid-transfer 1-like	leaf	2,47	
			cvCatherina.12044	ppa023836m	Non-specific lipid-transfer 1-like	leaf	1,12	
			cvCatherina.4632	ppa013464m	Non-specific lipid-transfer 8-like	leaf	-4,06	
		LTPs	GF677_6936	ppa014695m	Lipid-transfer DIR1	root	-1,93	
			GF677_20656	ppa021237m	Lipid-transfer DIR1	root	-2,64	
			GF677_11835	ppa016471m	Lipid-transfer DIR1	root	-3,00	
			cvCatherina.1090	ppa013722m	Lipid-transfer DIR1	leaf	-2,73	
		ABC	GF677_2380	ppa002351m	ABC transporter G family member 21	root	2,75	
			GF677_19512	ppa000236m	ABC transporter G family member 32	root	2,14	
			GF677_5054	ppa016652m	ABC transporter G family member 25	root	1,41	
			GF677_12006	ppa022260m	ABC transporter C family member 3-like	root	-0,56	
Cell wall	Extension		GF677_12964	ppa010376m	Expansin-like B1	root	1,91	
			cvCatherina.12041	ppa010314m	Expansin-A4-like precursor	leaf	-1,10	
			cvCatherina.17131	ppa010171m	Expansin-like A2	leaf	-1,23	
	Degradation		GF677_2000	ppa003156m	U-box domain-containing 19-like	root	-1,62	
			GF677_13579	ppa004653m	Endoglucanase 1	root	-2,64	
			**GF677_2725**	**ppa023604m**	**Lignin-forming anionic peroxidase 4-like**	**root**	**-6.15**	
			**cvCatherina.370**	**ppa005761m**	**Pectate lyase 1 related**	**leaf**	**-0,99**	
			**cvCatherina.5855**	**ppa011607m**	**Pectin methylesterase inhibitor**	**leaf**	**-3.53**	

Table S3 (B) Functional proteins (continued).

**Functions**		**Gene ID**	**ID in GDR**	**Annotation**	**Tissues**	**Log$_2$FC**	
Transport	General transporters	GF677_20074	ppa010996m	Vacuolar iron transporter homolog 4-like	root	1,66	
		GF677_12537	ppa025037m	Probable inorganic phosphate transporter 1-9	root	1,26	
		GF677_7237	ppa004254m	Glycerol-3-phosphate transporter 1	root	1,23	
		GF677_4246	ppa002542m	Sulfate transporter 1.3 like	root	1,17	
		GF677_21626	ppa003959m	Inorganic phosphate transporter 1-4	root	0,78	
		GF677_16142	ppa001860m	Nucleobase-ascorbate transporter 11 isoform X1	root	0,72	
		GF677_5963	ppa017165m	Bidirectional sugar transporter SWEET17-like	root	-1,13	
		GF677_5742	ppa027079m	GABA transporter 1-like	root	-1,16	
		GF677_15477	ppa021664m	Nitrate transporter	root	-1,47	
		GF677_10035	ppa001984m	Oligopeptide transporter 3	root	-1,71	
		GF677_11103	ppa025144m	Organic cation carnitine transporter 3-like	root	-2,03	
		GF677_13549	ppa022258m	Aluminum-activated malate transporter 10-like	root	-2,03	
		cvCatherina.1306	ppa016701m	Potassium transporter 5-like	leaf	-1.80e+308	**
		cvCatherina.5846	ppa004254m	Glycerol-3-phosphate transporter 1	leaf	1,86	
		cvCatherina.17465	ppa003959m	Inorganic phosphate transporter 1-4	leaf	1,14	
		cvCatherina.7429	ppa003996m	Glucose transporter 1	leaf	0,55	
		cvCatherina.5372	ppa003746m	Vacuolar amino acid transporter 1	leaf	-0,94	
		cvCatherina.8185	ppa001984m	Oligopeptide transporter 3	leaf	-1,34	
	Water transporter	**cvCatherina.12386**	**ppa010367m**	**Aquaporin TIP1.2**	**leaf**	**-3,13**	
LEA	Late embryogenesis	GF677_18885	ppa005514m	Dehydrin Rab 18	root	4,83	
Chloroplast	Chloroplastic membrane	**cvCatherina.15894**	**ppa006453m**	**Monogalactosyldiacylglycerol synthase 2, chloroplastic (MGD2)**	**leaf**	**2,66**	
Phosphate starvation	SPX domain	GF677_6071	ppa010111m	SPX domain-containing 3	root	2,31	
		GF677_19583	ppa009473m	SPX domain-containing 1-like	root	2,25	
		**cvCatherina.15841**	**ppa009473m**	**SPX domain-containing 1-like**	**leaf**	**3,40**	
		cvCatherina.7759	ppa008419m	SPX domain-containing 4	leaf	1,86	
		cvCatherina.3224	ppa009366m	SPX domain-containing 1-like	leaf	0,91	
	Purple acid phosphatase	GF677_14452	ppa008456m	Purple acid phosphatase 17	root	2,94	
		GF677_438	ppa003722m	Purple acid phosphatase 23	root	1,60	
		**cvCatherina.11767**	**ppa008418m**	**Purple acid phosphatase 17**	**leaf**	**4,59**	
		cvCatherina.374	ppa003722m	Purple acid phosphatase 23	leaf	1,68	
		cvCatherina.2459	ppa005293m	Purple acid phosphatase 2	leaf	1,55	
		cvCatherina.7049	ppa014823m	Probable inactive purple acid phosphatase 16	leaf	1,54	
		cvCatherina.4274	ppa002700m	Probable inactive purple acid phosphatase 27	leaf	1,28	
		cvCatherina.5326	ppa006786m	Probable inactive purple acid phosphatase 29	leaf	1,14	
