# Supplementary material for: Transcriptional Responses in Root and Leaf of Prunus persica under Drought Stress Using RNA Sequencing
Source: Front Plant Sci. 2016 Nov 23;7:1715. doi: 10.3389/fpls.2016.01715 (PMC5120087; doi:10.3389/fpls.2016.01715)
Supplement: Supplementary file 4 [file Table_4.PDF]

Table S4. Major classes of KEGG metabolic pathways identified, with the number of enzymes and differentially expressed genes (DEGs) involved in each pathway. The Gene ID is the same as that deposited in the ENA. The color bar indicates the transition of the expression pattern in terms of Log_2FC from upregulation (red squares) to downregulation (blue squares).The dark blue pattern with stars corresponds to genes uniquely expressed in the leaves of the control plants. The pathways involving at least two DEGs are represented in bold and illustrated in Figure S6. The fold change (FC) was calculated as the ratio between the drought-stressed and control plants.

A- Roots

Classes	Pathways	Pathway ID	N° of enzymes	N° of genes	Gene ID	Log_2FC
**Carbohydrate metabolism**	**Starch and sucrose metabolism**	map00500	3	4	GF677_13579	-2.64
					GF677_18434	2.08
					GF677_22052	0.68
					GF677_18506	0.76
	**Inositol phosphate metabolism**	map00562	4	3	GF677_21039	1.35
					GF677_2691	-2.14
					GF677_19350	-1.79
	**Pyruvate metabolism**	map00620	2	3	GF677_11293	-2.96
					GF677_14079	-2.91
					GF677_16384	0.60
	**Amino sugar and nucleotide sugar metabolism**	map00520	5	4	GF677_22052	0.68
					GF677_8275	-1.87
					GF677_540	-2.62
					GF677_11964	-1.50
	**Glyoxylate and dicarboxylate metabolism**	map00630	1	3	GF677_605	-2.25
					GF677_606	-2.30
					GF677_8702	2.61
	Ascorbate and aldarate metabolism	map00053	1	1	GF677_19350	-1.79
	Glycolysis / Gluconeogenesis	map00010	1	1	GF677_19994	-3.78
	Fructose and mannose metabolism	map00051	2	1	GF677_8275	-1.87
	Galactose metabolism	map00052	1	1	GF677_17056	2.82
	Pentose and glucuronate interconversions	map00040	2	1	GF677_1972	-2.44
	Pentose phosphate pathway	map00030	1	1	GF677_19490	1.72
**Lipid metabolism**	**Steroid hormone biosynthesis**	map00140	2	4	GF677_14574	1.71
					GF677_17486	0.73
					GF677_721	-1.06
					GF677_5166	-1.24
	**Fatty acid degradation**	map00071	3	3	GF677_19994	-3.78
					GF677_14574	1.71
					GF677_17486	0.73
	**Fatty acid biosynthesis**	map00061	1	2	GF677_20005	-3.37
					GF677_20004	-3.05
	**Glycerophospholipid metabolism**	map00564	3	2	GF677_17948	1.59
					GF677_17117	2.66
	**Arachidonic acid metabolism**	map00590	2	2	GF677_14574	1.71
					GF677_17486	0.73
	**Linoleic acid metabolism**	map00591	1	2	GF677_14574	1.71
					GF677_17486	0.73
	**Biosynthesis of unsaturated fatty acids**	map01040	1	2	GF677_20005	-3.37
					GF677_20004	-3.05
	Glycerolipid metabolism	map00561	1	1	GF677_10190	0.74
	Ether lipid metabolism	map00565	1	1	GF677_17117	2.66
	Alpha-Linolenic acid metabolism	map00592	1	1	GF677_19994	-3.78
**Metabolism of amino acid**	**Cysteine and methionine metabolism**	map00270	7	5	GF677_13569	1.67
					GF677_3733	-1.76
					GF677_4609	-3.81
					GF677_19306	-3.92
					GF677_16881	0.91
	**Phenylalanine metabolism**	map00360	5	3	GF677_3733	-1.76
					GF677_4609	-3.81
					GF677_524	-4.22
	**Tyrosine metabolism**	map00350	5	3	GF677_19994	-3.78
					GF677_3733	-1.76
					GF677_4609	-3.81
	**Arginine and proline metabolism**	map00330	1	2	GF677_3733	-1.76
					GF677_4609	-3.81
	**Arginine biosynthesis**	map00220	1	2	GF677_3733	-1.76
					GF677_4609	-3.81
	**Lysine biosynthesis**	map00300	2	2	GF677_4609	-3.81
					GF677_3733	-1.76
	**Alanine, aspartate and glutamate metabolism**	map00250	2	3	GF677_3733	-1.76
					GF677_4609	-3.81
					GF677_16881	0.91
	**Histidine metabolism**	map00340	1	2	GF677_3733	-1.76
					GF677_4609	-3.81
	**Cyanoamino acid metabolism**	map00460	2	2	GF677_18434	2.08
					GF677_22052	0.68
	**Tryptophan metabolism**	map00380	1	2	GF677_14574	1.71
					GF677_17486	0.73
	**Glycine, serine and threonine metabolism**	map00260	3	3	GF677_19994	-3.78
					GF677_8805	1.56
					GF677_16881	0.91
	**Phenylalanine, tyrosine and tryptophan biosynthesis**	map00400	4	2	GF677_3733	-1.76
					GF677_4609	-3.81
	Glutathione metabolism	map00480	1	1	GF677_14079	-2.91
	Selenocompound metabolism	map00450	2	1	GF677_13569	1.67

Table S4 (A) Roots (Continued).

Classes	Pathways	Pathway ID	N° of enzymes	N° of genes	Gene ID	Log_2FC
**Xenobiotics biodegradation and metabolism**	**Aminobenzoate degradation**	map00627	4	9	GF677_438	1.60
					GF677_15114	4.25
					GF677_16384	0.60
					GF677_14574	1.71
					GF677_17486	0.73
					GF677_19254	3.13
					GF677_14452	2.94
					GF677_2691	-2.14
					GF677_19391	1.85
	**Drug metabolism - cytochrome P450**	map00982	4	5	GF677_14079	-2.91
					GF677_19994	-3.78
					GF677_14574	1.71
					GF677_17486	0.73
					GF677_13110	-1.81
	**Drug metabolism - other enzymes**	map00983	1	2	GF677_10190	0.74
					GF677_1287	1.06
	**Steroid degradation**	map00984	1	2	GF677_721	-1.06
					GF677_5166	-1.24
	**Metabolism of xenobiotics by cytochrome P450**	map00980	3	4	GF677_14079	-2.91
					GF677_19994	-3.78
					GF677_14574	1.71
					GF677_17486	0.73
	Caprolactam degradation	map00930	1	1	GF677_14574	1.71
	Naphthalene degradation	map00626	1	1	GF677_19994	-3.78
	Chloroalkane and chloroalkene degradation	map00625	1	1	GF677_19994	-3.78
**Biosynthesis of other secondary metabolites**	**Phenylpropanoid biosynthesis**	map00940	3	9	GF677_18434	2.08
					GF677_22052	0.68
					GF677_2725	-6.15
					GF677_17639	-2.43
					GF677_18894	1.43
					GF677_16877	-1.79
					GF677_14803	-2.88
					GF677_15065	0.96
					GF677_524	-4.22
	**Flavonoid biosynthesis**	map00941	3	4	GF677_835	-2.28
					GF677_19946	-3.32
					GF677_721	-1.06
					GF677_524	-4.22
	**Caffeine metabolism**	map00232	1	2	GF677_14574	1.71
					GF677_17486	0.73
	**Isoquinoline alkaloid biosynthesis**	map00950	3	2	GF677_3733	-1.76
					GF677_4609	-3.81
	**Tropane, piperidine and pyridine alkaloid biosynthesis**	map00960	4	2	GF677_3733	-1.76
					GF677_4609	-3.81
	Stilbenoid, diarylheptanoid and gingerol biosynthesis	map00945	1	1	GF677_524	-4.22
	Isoflavonoid biosynthesis	map00943	1	1	GF677_12111	-3.74
**Metabolism of cofactors and vitamins**	**Thiamine metabolism**	map00730	1	5	GF677_2380	2.75
					GF677_12006	-0.56
					GF677_5054	1.41
					GF677_21543	1.22
					GF677_3912	0.88
	**Ubiquinone and other terpenoid-quinone biosynthesis**	map00130	2	3	GF677_3733	-1.76
					GF677_4609	-3.81
					GF677_524	-4.22
	**Retinol metabolism**	map00830	3	3	GF677_14574	1.71
					GF677_19994	-3.78
					GF677_17486	0.73
	Porphyrin and chlorophyll metabolism	map00860	1	1	GF677_19490	1.72
**Nucleotide metabolism**	**Purine metabolism**	map00230	2	5	GF677_2380	2.75
					GF677_12006	-0.56
					GF677_3912	0.88
					GF677_5054	1.41
					GF677_21543	1.22
**Energy metabolism**	**Carbon fixation in photosynthetic organisms**	map00710	2	5	GF677_605	-2.25
					GF677_606	-2.30
					GF677_8702	2.61
					GF677_4609	-3.81
					GF677_3733	-1.76
	Methane metabolism	map00680	1	1	GF677_8805	1.56
	Sulfur metabolism	map00920	1	1	GF677_13569	1.67
	Oxidative phosphorylation	map00190	1	1	GF677_13134	2.02
**Environmental information processing, signal transduction**	Phosphatidylinositol signaling system (Ph signal system)	map04070	2	2	GF677_2691	-2.14
					GF677_21039	1.35
**Metabolism of terpenoids and polyketides**	Carotenoid biosynthesis	map00906	1	1	GF677_18716	1.46
	Diterpenoid biosynthesis	map00904	1	1	GF677_835	-2.28
	Terpenoid backbone biosynthesis	map00900	2	1	GF677_14922	-1.16

Table S4 (B)

B- Leaves

Classes	Pathways	Pathway ID	N °of enzymes	N° of genes	Gene ID	Log_2FC	
**Carbohydrate metabolism**	**Pyruvate metabolism**	map00620	1	4	cvCatherina.17367	-1.80e+308	**
					cvCatherina.1098	-1.80e+308	**
					cvCatherina.9155	1.69	
					cvCatherina.11474	-1.80e+308	**
	**Glyoxylate and dicarboxylate metabolism**	map00630	1	2	cvCatherina.6446	-1.98	
					cvCatherina.11416	-4.80	
	**Ascorbate and aldarate metabolism**	map00053	1	2	cvCatherina.12438	1.36	
					cvCatherina.13321	1.22	
	**Pentose and glucuronate interconversions**	map00040	3	2	cvCatherina.676	1.12	
					cvCatherina.370	-0.99	
	Glycolysis / Gluconeogenesis	map00010	1	1	cvCatherina.15207	0.79	
	Amino sugar and nucleotide sugar metabolism	map00520	1	1	cvCatherina.2749	1.78	
	Citrate cycle (TCA cycle)	map00020	1	1	cvCatherina.6267	-0.37	
**Lipid metabolism**	**Glycerophospholipid metabolism**	map00564	4	4	cvCatherina.11558	1.07	
					cvCatherina.8096	3.54	
					cvCatherina.7529	0.50	
					cvCatherina.9162	-0.72	
	**Glycerolipid metabolism**	map00561	2	2	cvCatherina.2749	1.78	
					cvCatherina.7529	0.50	
	Alpha-Linolenic acid metabolism	map00592	1	1	cvCatherina.9162	-0.72	
	Arachidonic acid metabolism	map00590	1	1	cvCatherina.9162	-0.72	
	Linoleic acid metabolism	map00591	1	1	cvCatherina.9162	-0.72	
	Steroid hormone biosynthesis	map00140	1	1	cvCatherina.4172	0.61	
	Ether lipid metabolism	map00565	1	1	cvCatherina.9162	-0.72	
**Metabolism of amino acid**	**Glutathione metabolism**	map00480	4	10	cvCatherina.17367	-1.80e+308	**
					cvCatherina.1098	-1.80e+308	**
					cvCatherina.11474	-1.80e+308	
					cvCatherina.6267	-0.37	
					cvCatherina.12893	0.48	
					cvCatherina.12438	1.36	
					cvCatherina.13321	1.22	
					cvCatherina.9155	1.69	
					cvCatherina.266	-1.80e+308	
					cvCatherina.13478	-1.80e+308	
	Taurine and hypotaurine metabolism	map00430	1	1	cvCatherina.12893	0.48	
	Tyrosine metabolism	map00350	1	1	cvCatherina.4172	0.61	
	Alanine, aspartate and glutamate metabolism	map00250	1	1	cvCatherina.11963	-1.14	
	Cyanoamino acid metabolism	map00460	1	1	cvCatherina.12893	0.48	
	Glycine, serine and threonine metabolism	map00260	1	1	cvCatherina.6955	-1.66	
	Phenylalanine, tyrosine and tryptophan biosynthesis	map00400	1	1	cvCatherina.6955	-1.66	
**Xenobiotics biodegradation and metabolism**	**Aminobenzoate degradation**	map00627	2	10	cvCatherina.6471	0.74	
					cvCatherina.4274	1.28	
					cvCatherina.2459	1.55	
					cvCatherina.374	1.68	
					cvCatherina.15676	0.68	
					cvCatherina.6526	2.61	
					cvCatherina.8503	0.32	
					cvCatherina.11767	4.59	
					cvCatherina.7049	1.54	
					cvCatherina.7411	0.97	
	**Drug metabolism - cytochrome P450**	map00982	1	6	cvCatherina.17367	-1.80e+308	**
					cvCatherina.1098	-1.80e+308	**
					cvCatherina.9155	1.69	
					cvCatherina.11474	-1.80e+308	**
					cvCatherina.266	-1.80e+308	**
					cvCatherina.13478	-1.80e+308	**
	**Drug metabolism - other enzymes**	map00983	1	3	cvCatherina.16793	-1.05	
					cvCatherina.17541	-1.46	
					cvCatherina.9162	-0.72	
	**Metabolism of xenobiotics by cytochrome P450**	map00980	1	6	cvCatherina.17367	-1.80e+308	**
					cvCatherina.1098	-1.80e+308	**
					cvCatherina.9155	1.69	
					cvCatherina.11474	-1.80e+308	**
					cvCatherina.266	-1.80e+308	**
					cvCatherina.13478	-1.80e+308	**
**Metabolism of cofactors and vitamins**	**Thiamine metabolism**	map00730	1	4	cvCatherina.15787	-7.22	
					cvCatherina.7027	-1.79	
					cvCatherina.3901	-0.43	
					cvCatherina.10835	-1.04	
	Porphyrin and chlorophyll metabolism	map00860	2	3	cvCatherina.17541	-1.46	
					cvCatherina.13605	2.35	
					cvCatherina.5689	2.82	
	Vitamin B6 metabolism	map00750	1	1	cvCatherina.6526	2.61	
**Biosynthesis of other secondary metabolites**	**Phenylpropanoid biosynthesis**	map00940	1	5	cvCatherina.3325	0.20	
					cvCatherina.12438	1.36	
					cvCatherina.13321	1.22	
					cvCatherina.5224	1.84	
					cvCatherina.473	-1.80e+308	**
	Betalain biosynthesis	map00965	1	1	cvCatherina.4172	0.61	
	Flavonoid biosynthesis	map00941	1	1	cvCatherina.17817	-0.77	
**Nucleotide metabolism**	**Purine metabolism**	map00230	2	4	cvCatherina.15787	-7.22	
					cvCatherina.7027	-1.79	
					cvCatherina.3901	-0.43	
					cvCatherina.10835	-1.04	
**Energy metabolism**	Nitrogen metabolism	map00910	2	1	cvCatherina.3411	-1.09	
	Oxidative phosphorylation	map00190	2	1	cvCatherina.2830	0.82	
	Carbon fixation pathways in prokaryotes	map00720	1	1	cvCatherina.6267	-0.37	
**Environmental information processing, signal transduction**	Phosphatidylinositol signaling system	map04070	1	1	cvCatherina.7529	0.50	
