# Supplementary material for: Transcriptional Responses in Root and Leaf of Prunus persica under Drought Stress Using RNA Sequencing
Source: Front Plant Sci. 2016 Nov 23;7:1715. doi: 10.3389/fpls.2016.01715 (PMC5120087; doi:10.3389/fpls.2016.01715)
Supplement: Supplementary file 5 [file Image_1.PDF]

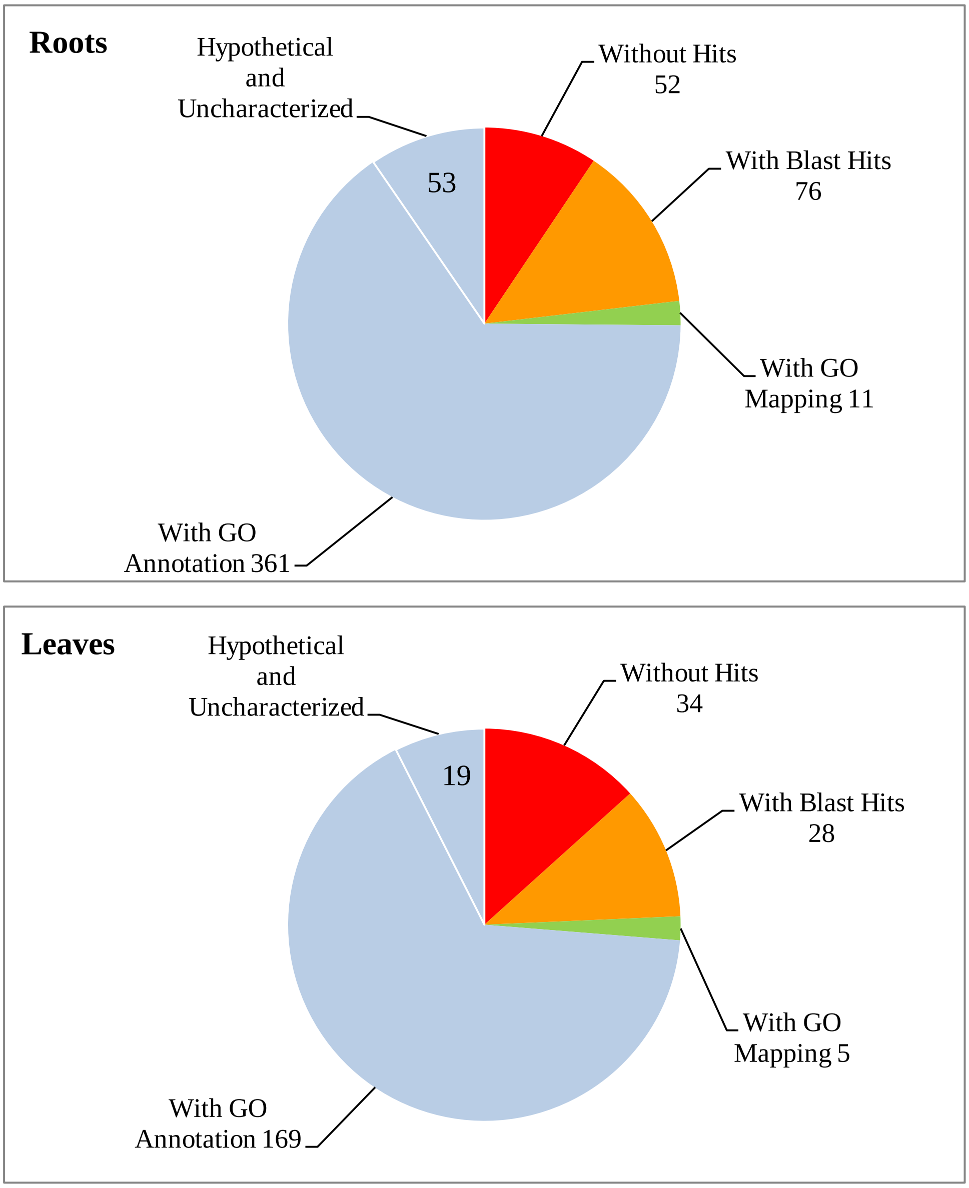

Figure S1 Annotation summary of differentially expressed genes (DEGs; *Q*-value < 0.01) in root and leaf tissues (GF677 rootstock budded with var. Catherina). GO mapping indicates genes that were mapped but not annotated.
