# Supplementary material for: Transcriptional Responses in Root and Leaf of Prunus persica under Drought Stress Using RNA Sequencing
Source: Front Plant Sci. 2016 Nov 23;7:1715. doi: 10.3389/fpls.2016.01715 (PMC5120087; doi:10.3389/fpls.2016.01715)
Supplement: Supplementary file 6 [file Image_2.PDF]

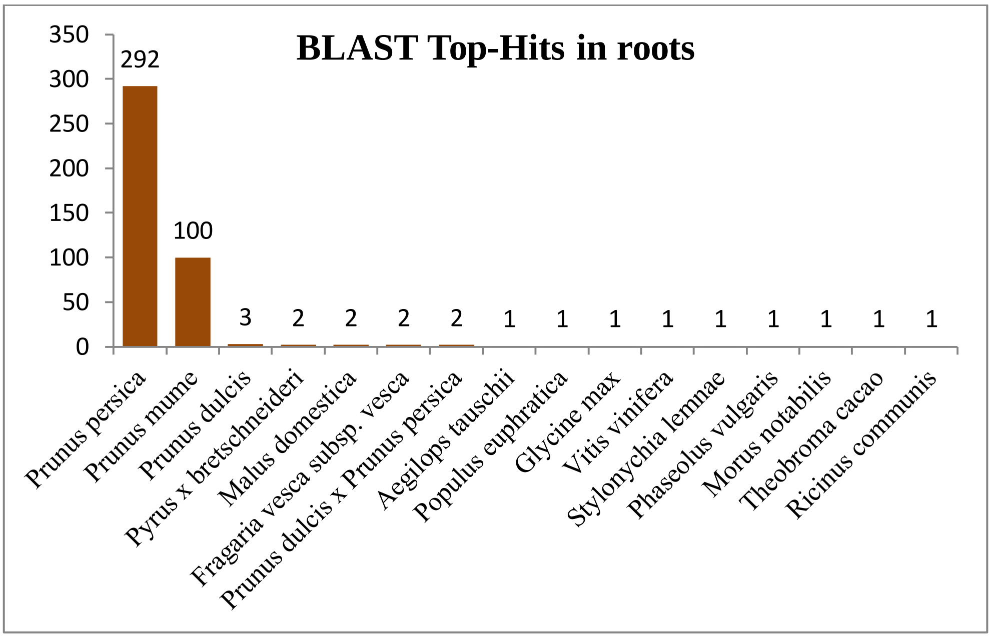

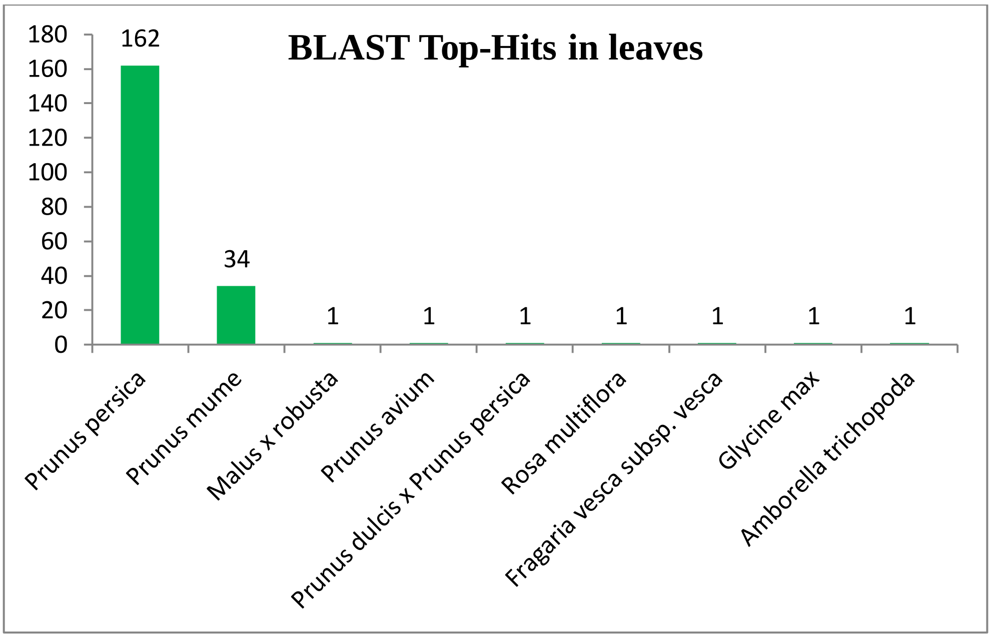

Figure S2 Species distribution according to the BLASTX top hits in roots (GF677 rootstock) and leaves (graft, var. Catherina).
