# Supplementary material for: Transcriptional Responses in Root and Leaf of Prunus persica under Drought Stress Using RNA Sequencing
Source: Front Plant Sci. 2016 Nov 23;7:1715. doi: 10.3389/fpls.2016.01715 (PMC5120087; doi:10.3389/fpls.2016.01715)
Supplement: Supplementary file 7 [file Image_3.PDF]

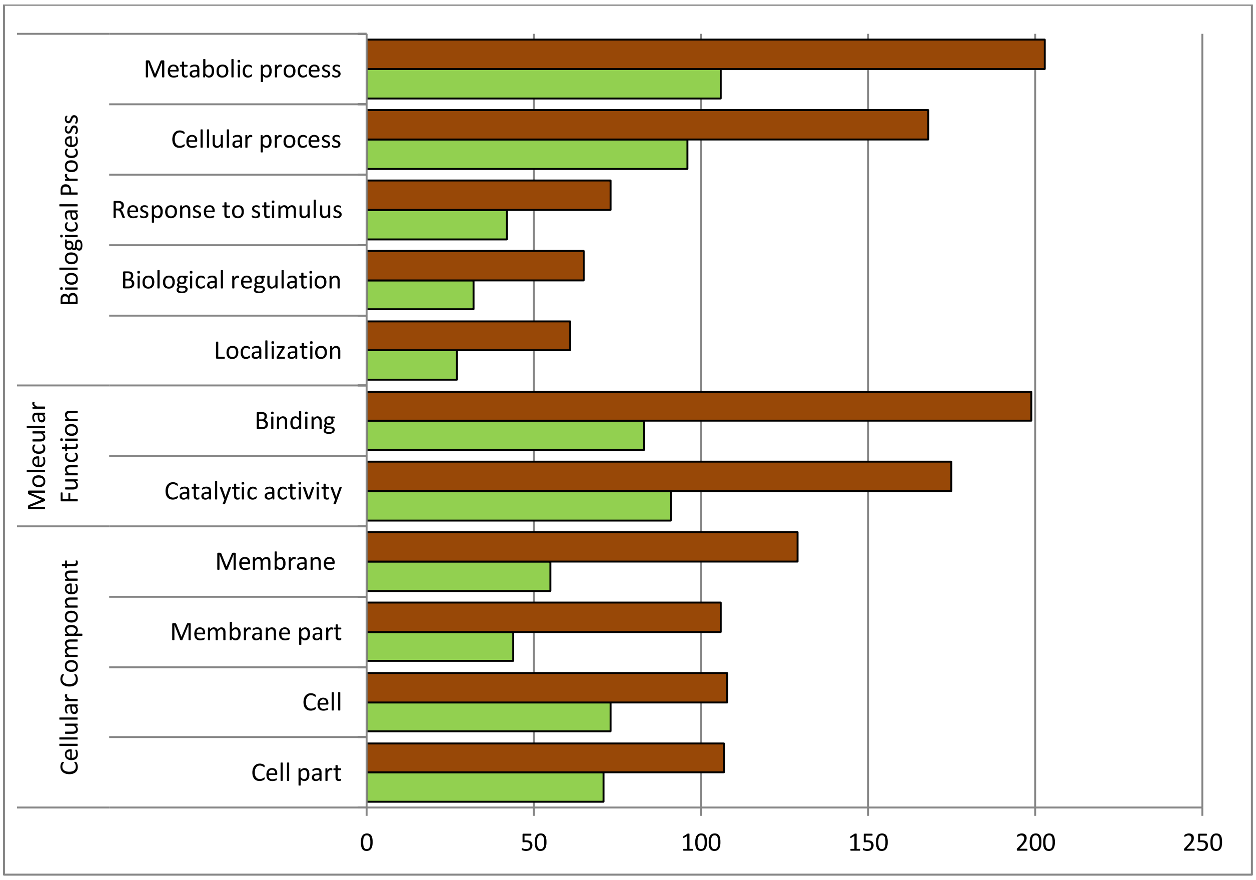

Figure S3 Most frequent gene ontology (GO) terms assigned in the three main categories: biological processes (BP), molecular functions (MF), and cellular components (CC). The *x*-axis indicates the number of differentially expressed genes (DEGs) in each category. Brown color corresponds to roots (GF677 rootstock) and green color to leaves (graft, var. Catherina).
