# Supplementary material for: Transcriptional Responses in Root and Leaf of Prunus persica under Drought Stress Using RNA Sequencing
Source: Front Plant Sci. 2016 Nov 23;7:1715. doi: 10.3389/fpls.2016.01715 (PMC5120087; doi:10.3389/fpls.2016.01715)
Supplement: Supplementary file 8 [file Image_4.PDF]

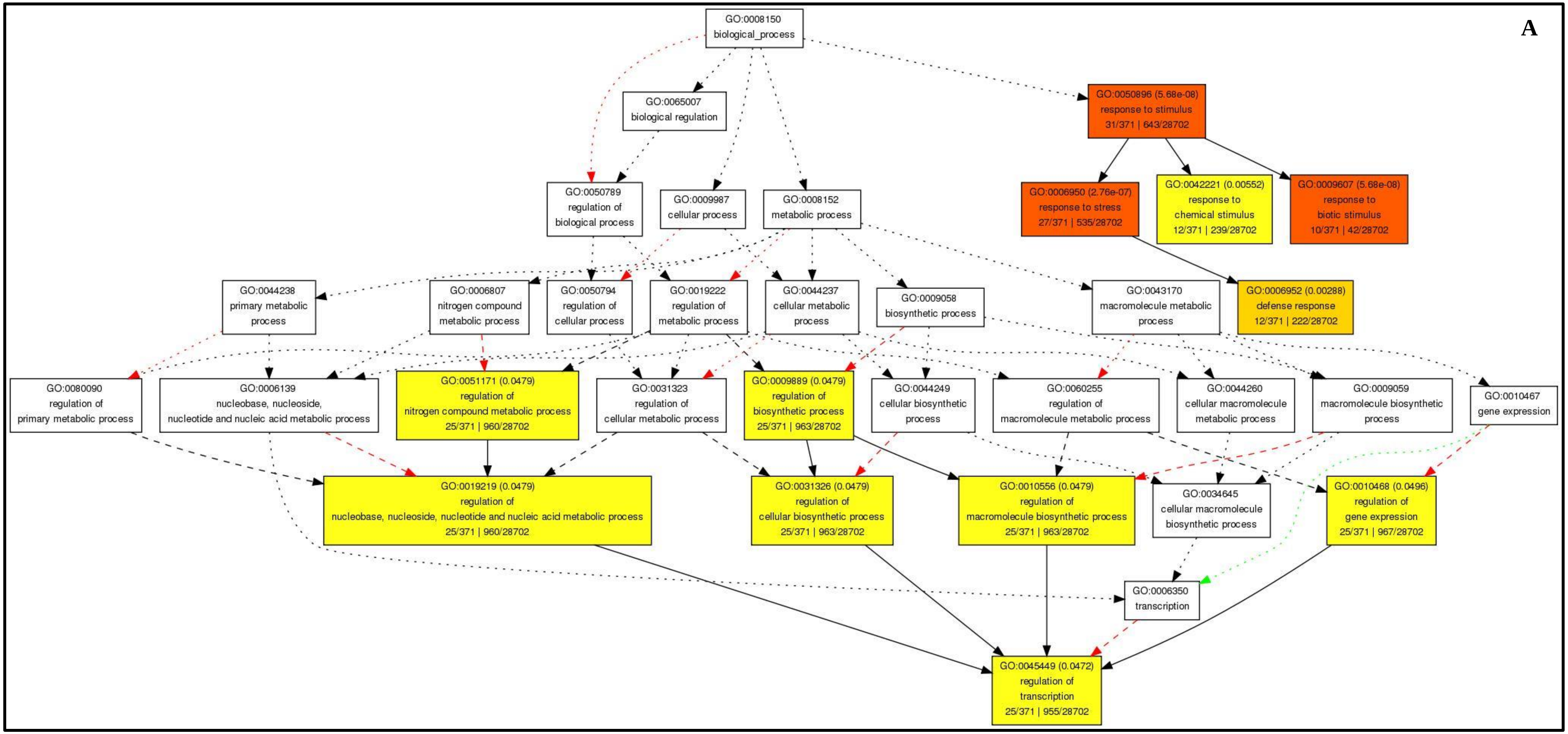
A
GO:0008150 biological_process
GO:0065007 biological regulation
GO:0050896 (5.68e-08) response to stimulus 31/371 | 643/28702
GO:0050789 regulation of biological process
GO:0009987 cellular process
GO:0008152 metabolic process
GO:0006950 (2.76e-07) response to stress 27/371 | 535/28702
GO:0042221 (0.00552) response to chemical stimulus 12/371 | 239/28702
GO:0009607 (5.68e-08) response to biotic stimulus 10/371 | 42/28702
GO:0044238 primary metabolic process
GO:0006807 nitrogen compound metabolic process
GO:0050794 regulation of cellular process
GO:0019222 regulation of metabolic process
GO:0044237 cellular metabolic process
GO:0009058 biosynthetic process
GO:0043170 macromolecule metabolic process
GO:0006952 (0.00288) defense response 12/371 | 222/28702
GO:0080090 regulation of primary metabolic process
GO:0006139 nucleobase, nucleoside, nucleotide and nucleic acid metabolic process
GO:0051171 (0.0479) regulation of nitrogen compound metabolic process 25/371 | 960/28702
GO:0031323 regulation of cellular metabolic process
GO:0009889 (0.0479) regulation of biosynthetic process 25/371 | 963/28702
GO:0044249 cellular biosynthetic process
GO:0060255 regulation of macromolecule metabolic process
GO:0044260 cellular macromolecule metabolic process
GO:0009059 macromolecule biosynthetic process
GO:0010467 gene expression
GO:0019219 (0.0479) regulation of nucleobase, nucleoside, nucleotide and nucleic acid metabolic process 25/371 | 960/28702
GO:0031326 (0.0479) regulation of cellular biosynthetic process 25/371 | 963/28702
GO:0010556 (0.0479) regulation of macromolecule biosynthetic process 25/371 | 963/28702
GO:0034645 cellular macromolecule biosynthetic process
GO:0010468 (0.0496) regulation of gene expression 25/371 | 967/28702
GO:0006350 transcription
GO:0045449 (0.0472) regulation of transcription 25/371 | 955/28702

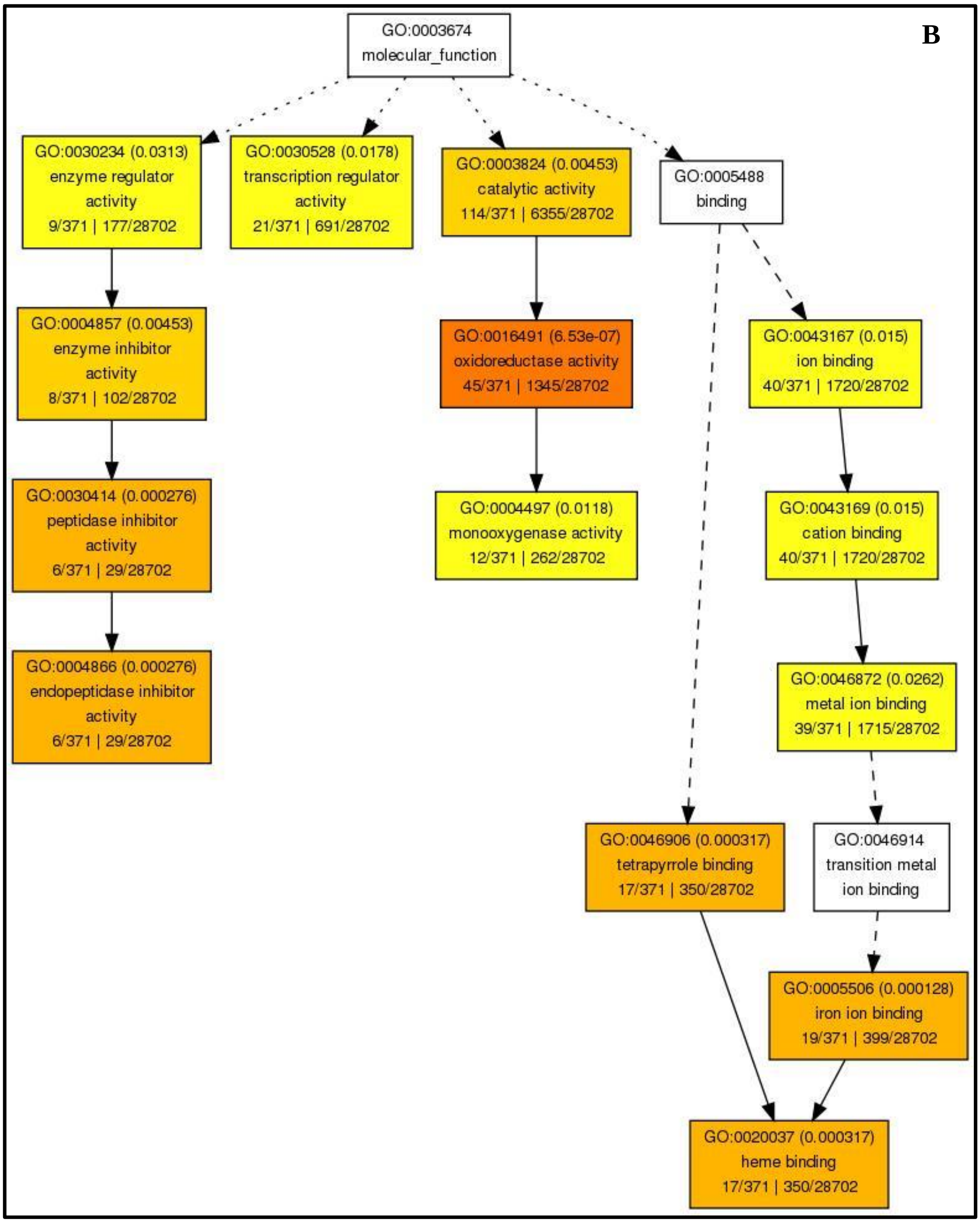

Figure S4 Significant gene ontology (GO) terms from biological processes (**A**) and molecular functions (**B**) enriched in roots (GF677 rootstock), after a singular enrichment analysis (SEA) in AgriGO. Each box indicates the GO number and full description with *Q*-values in parenthesis. The numbers on the left side indicate the fraction of DEG genes sharing each GO term. The numbers on the right side indicate the background numbers of genes associated with the GO term in the *P. persica* genome. The significance of the enrichment is displayed using a color scale from yellow to red. The white boxes show GO terms with adjusted *P*-values > 0.05. The differentially expressed genes (DEGs) involved in "Response to stimulus" (GO: 0050896) are magnified in Figure 4.
