# Supplementary material for: Transcriptional Responses in Root and Leaf of Prunus persica under Drought Stress Using RNA Sequencing
Source: Front Plant Sci. 2016 Nov 23;7:1715. doi: 10.3389/fpls.2016.01715 (PMC5120087; doi:10.3389/fpls.2016.01715)
Supplement: Supplementary file 9 [file Image_5.PDF]

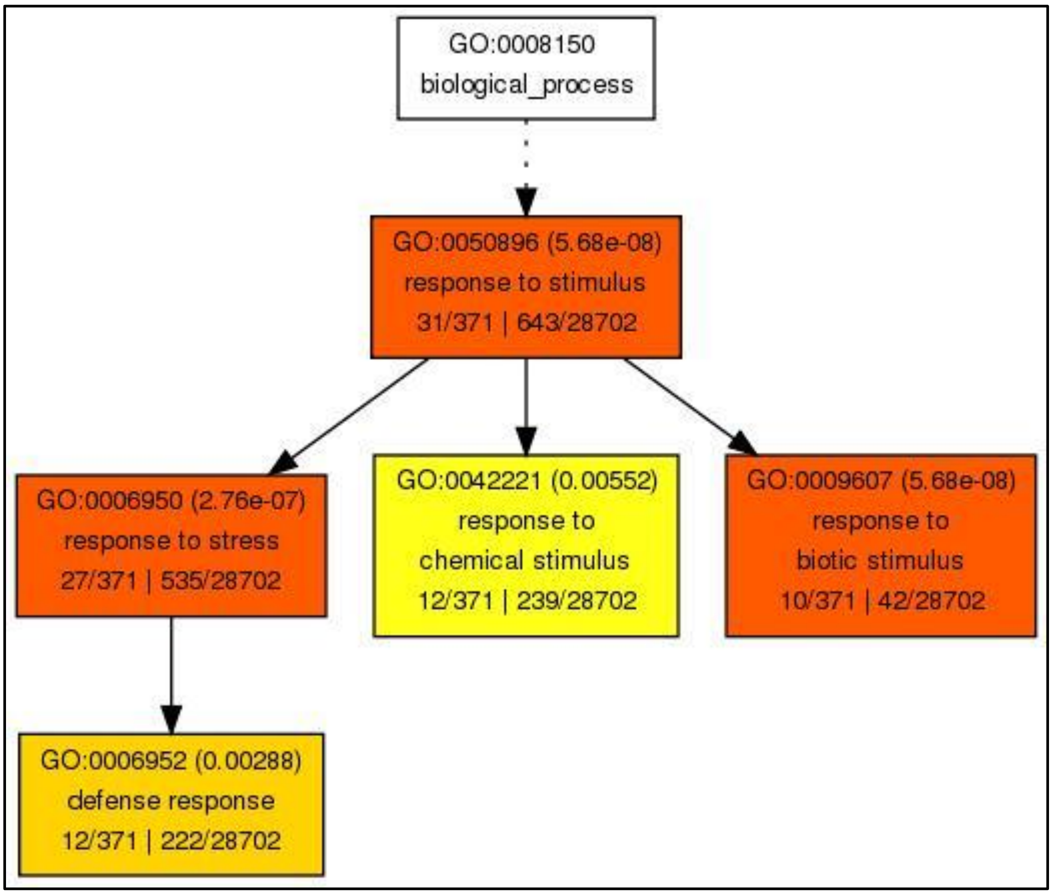

Figure S5 The top enriched gene ontology (GO) terms in the biological processes (BP) category in roots (GF677 rootstock) after a singular enrichment analysis (SEA) in AgriGO. Each box indicates the GO number and a full description, with the *Q*-value in parenthesis. The numbers on the left side indicate the fraction of DEGs sharing the GO term. The numbers in the right side indicate the background numbers of genes associated with the GO term in the *P. persica* genome. The significance of the enrichment is displayed using a color scale from yellow to red. The complete set of enriched GO terms is illustrated in Figure S4A.
