# Supplementary material for: Transcriptional Responses in Root and Leaf of Prunus persica under Drought Stress Using RNA Sequencing
Source: Front Plant Sci. 2016 Nov 23;7:1715. doi: 10.3389/fpls.2016.01715 (PMC5120087; doi:10.3389/fpls.2016.01715)
Supplement: Supplementary file 10 [file Image_6.PDF]

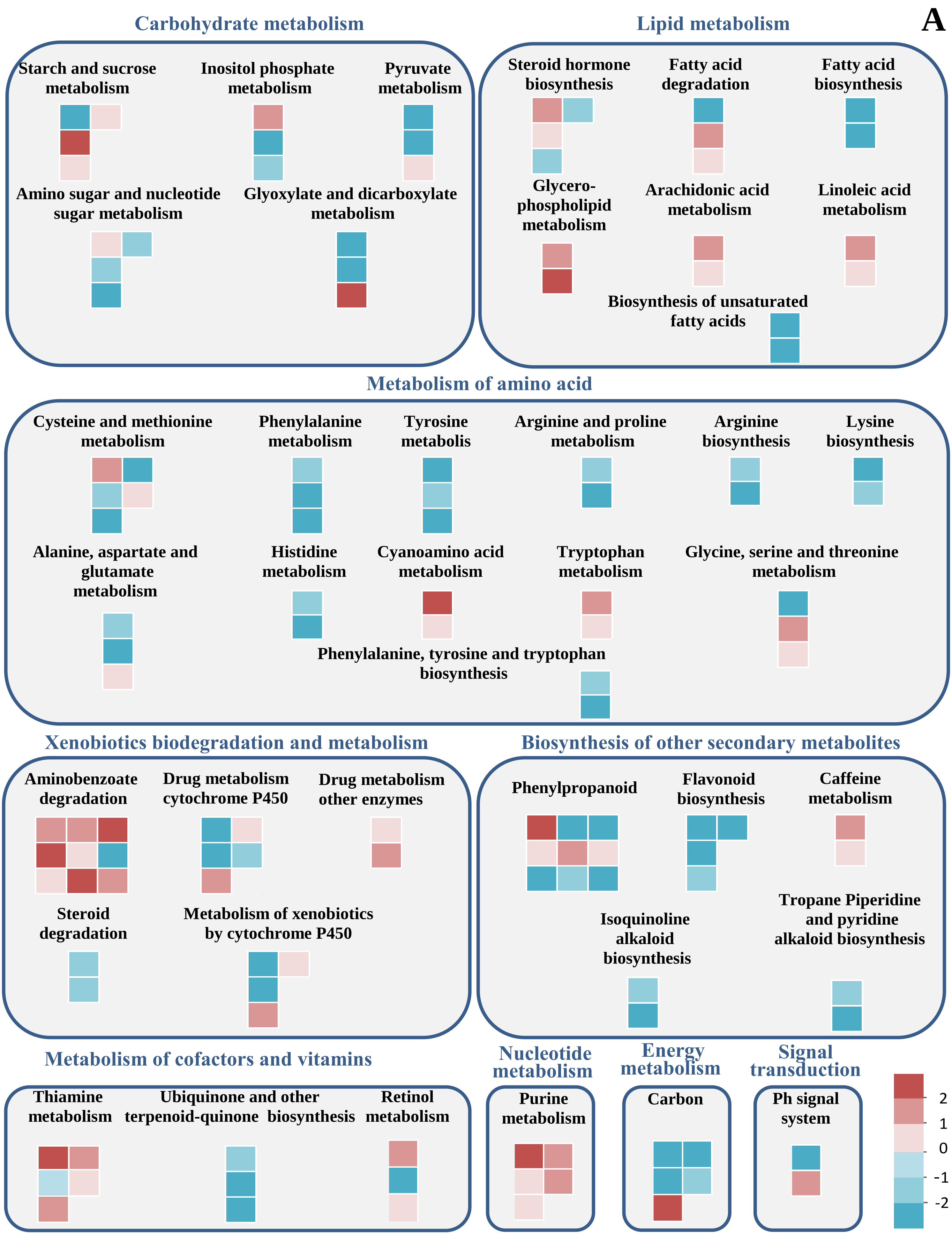
A
Carbohydrate metabolism
Starch and sucrose metabolism
Inositol phosphate metabolism
Pyruvate metabolism
Amino sugar and nucleotide sugar metabolism
Glyoxylate and dicarboxylate metabolism
Lipid metabolism
Steroid hormone biosynthesis
Fatty acid degradation
Fatty acid biosynthesis
Glycero-phospholipid metabolism
Arachidonic acid metabolism
Linoleic acid metabolism
Biosynthesis of unsaturated fatty acids
Metabolism of amino acid
Cysteine and methionine metabolism
Phenylalanine metabolism
Tyrosine metabolis
Arginine and proline metabolism
Arginine biosynthesis
Lysine biosynthesis
Alanine, aspartate and glutamate metabolism
Histidine metabolism
Cyanoamino acid metabolism
Tryptophan metabolism
Glycine, serine and threonine metabolism
Phenylalanine, tyrosine and tryptophan biosynthesis
Xenobiotics biodegradation and metabolism
Aminobenzoate degradation
Drug metabolism cytochrome P450
Drug metabolism other enzymes
Steroid degradation
Metabolism of xenobiotics by cytochrome P450
Biosynthesis of other secondary metabolites
Phenylpropanoid
Flavonoid biosynthesis
Caffeine metabolism
Isoquinoline alkaloid biosynthesis
Tropane Piperidine and pyridine alkaloid biosynthesis
Metabolism of cofactors and vitamins
Thiamine metabolism
Ubiquinone and other terpenoid-quinone biosynthesis
Retinol metabolism
Nucleotide metabolism
Purine metabolism
Energy metabolism
Carbon
Signal transduction
Ph signal system
2
1
0
-1
-2

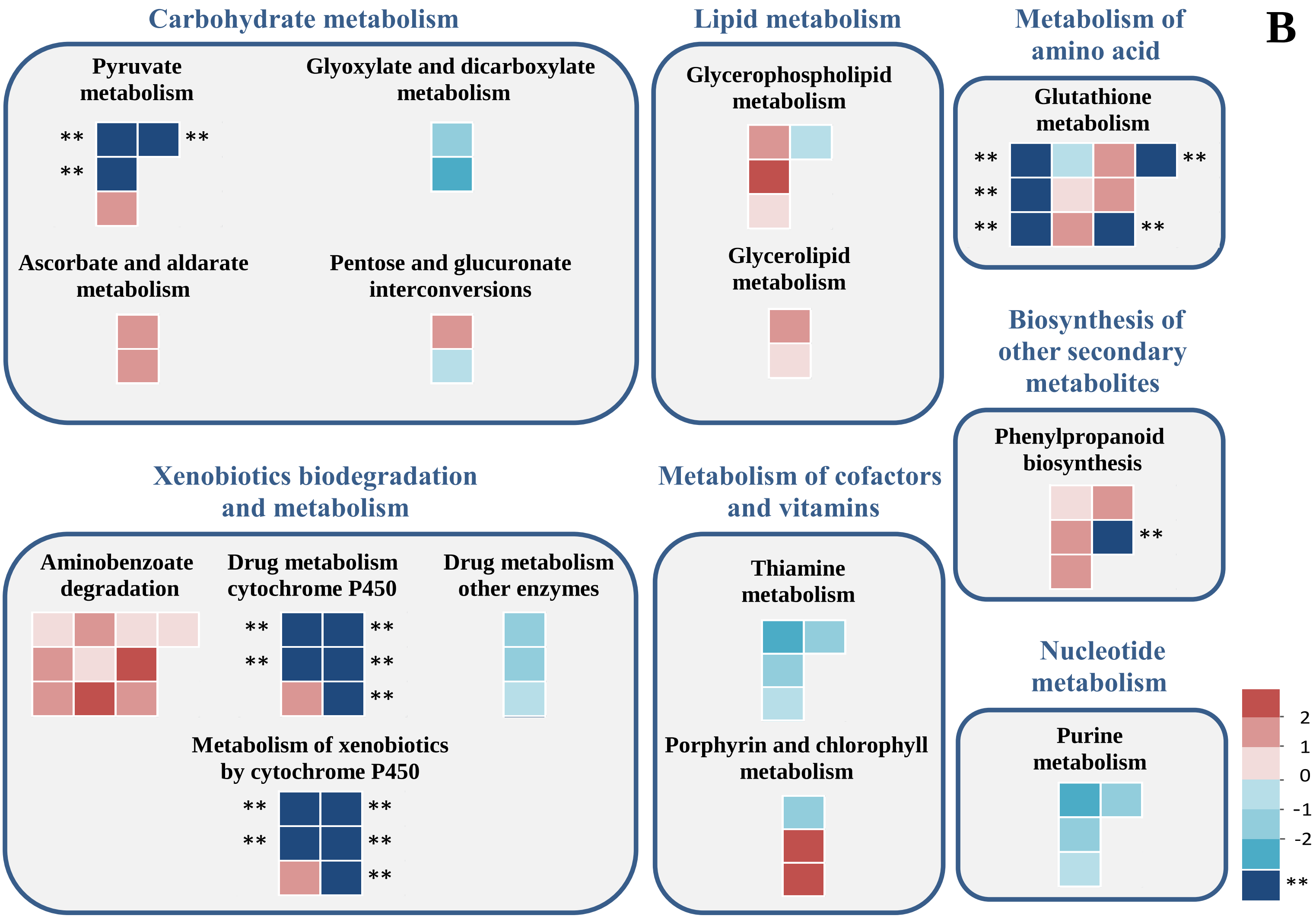

Figure S6 Major KEGG classes and the levels of expression of differentially expressed (DEGs) involved in each pathway. (**A**) Roots (GF677 rootstock). (**B**) Leaves (graft, var. Catherina. The scale bar on the right represents the observed changes in expression in terms of Log_2FC from upregulation (red squares) to downregulation (blue squares). The dark blue pattern with stars indicates genes uniquely expressed in leaves in the control group. The fold change was calculated as the ratio between the drought-stressed and control plants. Details are provided in Table S4.
